# BMP6/TAZ-Hippo signaling modulates angiogenesis and endothelial cell response to VEGF

**DOI:** 10.1007/s10456-020-09748-4

**Published:** 2020-10-06

**Authors:** H. H. Pulkkinen, M. Kiema, J. P. Lappalainen, A. Toropainen, M. Beter, A. Tirronen, L. Holappa, H. Niskanen, M. U. Kaikkonen, S. Ylä-Herttuala, Johanna P. Laakkonen

**Affiliations:** 1 A.I. Virtanen Institute for Molecular Sciences, University of Eastern Finland, Kuopio, Finland; 2 Department of Clinical Chemistry, University of Eastern Finland and Eastern Finland Laboratory Centre, Kuopio, Finland; 3 Science Service Center, Kuopio University Hospital, Kuopio, Finland; 4 Gene Therapy Unit, Kuopio University Hospital, Kuopio, Finland

**Keywords:** Angiogenesis, Bone morphogenetic protein, BMP2, BMP6, Hippo signaling pathway, Vascular endothelial growth factor, VEGF, VEGFR2

## Abstract

The BMP/TGFβ-Smad, Notch and VEGF signaling guides formation of endothelial tip and stalk cells. However, the crosstalk of bone morphogenetic proteins (BMPs) and vascular endothelial growth factor receptor 2 (VEGFR2) signaling has remained largely unknown. We demonstrate that BMP family members regulate VEGFR2 and Notch signaling, and act via TAZ-Hippo signaling pathway. BMPs were found to be regulated after VEGF gene transfer in C57/Bl6 mice and in a porcine myocardial ischemia model. BMPs 2/4/6 were identified as endothelium-specific targets of VEGF. BMP2 modulated VEGF-mediated endothelial sprouting via Delta like Canonical Notch Ligand 4 (DLL4). BMP6 modulated VEGF signaling by regulating VEGFR2 expression and acted via Hippo signaling effector TAZ, known to regulate cell survival/proliferation, and to be dysregulated in cancer. In a matrigel plug assay in nude mice BMP6 was further demonstrated to induce angiogenesis. BMP6 is the first member of BMP family found to directly regulate both Hippo signaling and neovessel formation. It may thus serve as a target in pro/anti-angiogenic therapies.

**Electronic supplementary material:**

The online version of this article (10.1007/s10456-020-09748-4) contains supplementary material, which is available to authorized users.

## Introduction

Aberrant vascular endothelial growth factor 2 (VEGFR2) signaling and increased VEGF expression has been connected to pathological angiogenesis in various vascular diseases and cancer [1–4]. VEGF-mediated gene transfer has also been used to induce vascular growth in myocardium and skeletal muscle to treat ischemia [5, 6]. Although next-generation sequencing analyses of VEGF-induced effects in endothelial cells have been performed [7–9], the crosstalk of multiple cell types, and other growth factor pathways regulating VEGF-induced angiogenesis are still poorly understood.

Emerging knowledge supports the role of bone morphogenetic proteins (BMPs) in vascular homeostasis and angiogenesis. Dysfunctional BMP signaling is involved in various vascular disorders, such as hereditary hemorrhagic telangiectasia, cerebral cavernous malformation, pulmonary arterial hypertension and atherosclerosis [10–12]. Concomitantly, BMP2/4, BMP receptors ALK1, ALK2, ALK3, or BMPR2 mouse knockouts lead to severe cardiovascular defects and embryonic lethality [13–16]. Specific BMP members have been shown to either stimulate or inhibit vessel formation. BMP2, -4, -6 and -7 are suggested to be pro-angiogenic, whereas BMP9 and BMP13 have anti-angiogenic effects [10, 17−21]. Generally, BMPs bind to two types of receptors: BMPRI (ALK1-3 and ALK6) and BMPRII (incl. BMPR2, ACVR2, ACVR2B), which form a heteromeric complex. Additionally, repulsive guidance molecules (RGMA-C), gremlin 1, BMP and activin membrane-bound inhibitor (BAMBI), and endoglin act as co-receptors [22, 23]. BMP receptors can initiate Smad signaling cascades, as well as phosphatidylinositol 3-kinase (PI3K) and mitogen-activated protein kinase (MAPK) signaling pathways.

Our findings demonstrate that (i) several BMPs are regulated after systemic VEGF-induced angiogenesis and in normoxic endothelial cells, (ii) BMPs are regulated after acute myocardial ischemia, and in hypoxic endothelial cells, (iii) BMP2 and BMP6 synergistically modulate VEGF-induced endothelial cell sprouting via regulating VEGFR, Notch or TAZ-Hippo signaling, and (iv) BMP6 protein is pro-angiogenic in vivo. Thus, BMPs are potential targets to modulate formation of vasculature in pro- and anti-angiogenic therapies.

## Materials and methods

Detailed methods section is available in the Supplemental Methods and Materials in the Major Resources Table.

### Materials

Human umbilical vein endothelial cells (HUVECs) were isolated from umbilical cords as previously described [24], and cultured in Endothelial Cell Growth medium (Promocell, Heidelberg, Germany) on fibronectin-gelatin coated surfaces (10 µg/ml, 0.05%; Sigma-Aldrich, St. Louis, MO). Passage < 6 was used for the experiments. Human lung primary fibroblasts (HPF) were purchased from Promocell and cultured in DMEM (10% FBS). Passage < 11 was used for the experiments. Silencer Select siRNAs against TAZ, TEAD2, BMP2, BMP6 and two control siRNAs were purchased from Thermo Fisher Scientific (Waltham, MA). For detailed information on the materials please see the Major Resources Table in the Supplemental Material.

### Experimental animals

Gene transfer experiments were performed with 8–12 weeks old male C57/Bl6 mice (Harlan Laboratories, Indianapolis, IN). Mice were injected via tail vein under isoflurane anesthesia with adenovirus expressing VEGF-A_165_ under CMV promoter (serotype 5, 1.4 × 10^10^ vp). Empty adenovirus without a transgene containing only the CMV promoter was used as a control. Mice were sacrificed 6 days after the gene transfer. After PBS perfusion, tissues were harvested and snap frozen in liquid nitrogen for RT-qPCR and RNA-sequencing. For imaging purposes tissues were fixed in 4% PFA in PBS for 4 h, embedded in paraffin and sectioned for immunohistochemical stainings. Matrigel plug angiogenesis assay was performed on 6-week-old Hsd:Athymic Nude-*Foxn1*^nu^ mice (Envigo, Indianapolis, IN). Mice were injected subcutaneously in their flank with 350 µl of growth factor reduced matrigel (Corning Life Sciences, Tewksbury, MA) containing 1 µM of sphingosine-1-phosphate (:S1P, Enzo Life Sciences, Farmingdale, NY) alone or together with 1.75 µg of human BMP6 recombinant protein (R&D Systems, Minneapolis, MN). Plugs contained 0.25 mg/ml fatty acid free bovine serum albumin (BSA, Biowest, Nuaillé, France) and DMEM with high glucose (Sigma Aldrich) leading to final matrigel protein concentration of 7 mg/ml. Each mouse had a control plug with only S1P and a plug with recombinant protein/s (*n* = 6 mice/treatment). Mice were sacrificed 7 days later, and the plugs were resected from surrounding tissues and fixed in 4% PFA in PBS for 4 h at RT. The plugs were embedded in paraffin in two parts to create cross-sections of both ends of the plug. The sections were labeled with HE or CD31-antibody. Imaging for the quantitation of cell nuclei area was performed with Leica Thunder 3D Tissue Imager from the whole plug area (× 20 objective) and of CD31-positive area with Eclipse Ni-E Nikon microscope from the plug edge areas (*n* = 8 images/plug, 20×/0.5 Plan Fluor objectives). Quantitative analysis were performed with NIS-Elements Analysis software.

### RT-qPCR

 Confluent cultures of HUVECs were washed with PBS, followed by starvation of cells for 16 h with EGM medium supplemented with 0.5% FBS. With siRNA experiments, HUVECs were transfected with 5 or 10 nM siRNA oligonucleotides using oligofectamine for 48 h (Life Technologies, Carlsbad, CA). Protein stimulations were performed with 50 ng/ml (0.5 h, 1 h, 2 h, 4 h, 7 h) or 100 ng/ml (7 h) of VEGF-A_165_ (VEGF) and 100 ng/ml of BMP2 or BMP6 (7 h; R&D Systems). Tissue samples were collected from VEGF or control virus-treated mice and homogenized with tissue homogenizer (Qiagen, Hilden, Germany). RNA was extracted with RNeasy Mini Kit (Qiagen) or with Tri-reagent (Molecular Research Center, Cincinnati, OH) according to the manufacturer’s instructions. Total RNA was reverse transcribed into cDNA using random hexamers and RevertAID reverse transcriptase (Thermo Fisher Scientific). Quantitative measurements of mRNA levels were performed using the Assays-on-Demand gene expression products (please see the Major Resources Table in the Supplemental Material) with StepOnePlus Real-Time PCR System (Applied Biosystems, Foster City, CA). Amplification of beta-2 microglobulin (B2M) was used as an endogenous control with human endothelial cells and peptidylprolyl isomerase A (PPIA) with mouse tissues. siRNA transfection efficiencies were 67.8% for TAZ, 74.6% for TEAD2, 68.3% for BMP2 and 83.0% for BMP6 detected by RT-qPCR. Decreased expression of TAZ was accordingly detected by western blot (Supplementary Fig. 4c).

### NGS experiments

For detailed information see Supplemental Materials and Methods. Briefly, RNA was extracted from the mice liver tissue at d6. After enrichment, RNA was fragmented and purified. Poly(A)-tailing and cDNA synthesis was performed as described [25]. For reverse transcription an oligo allowing custom barcoding during the final amplification was used. Exonuclease I (New England Biolabs, Ipswich, MA) was used to catalyze the removal of excess oligos. The DNA–RNA hybrid was further purified (Zymo Research Corporation, Irvine, CA), treated with RNaseH and circularized using CircLigase (Epicentre, Madison, WI). Libraries were amplified, purified and sequenced on HiSeq 2000 according to the manufacturer’s instructions (GeneCore, EMBL, Heidelberg, Germany). RNA-Seq data pre-processing and analysis were performed as described previously [26].

RNA-Seq was mapped using TopHat (v2.0.7). Each sequencing experiment was normalized to the total of 10^7^ uniquely mapped tags and visualized by preparing custom tracks for the UCSC Genome browser. The following thresholds were used: FDR < 0.05, RPKM > 0.5, log fold changes > 1.0 and < − 1.0. Gene expression tags were normalized, log transformed and centered to − 1 to 1 prior to clustering. Clustering and heatmaps were generated with Cluster 3.0 and Java Treeview softwares using hierarchical clustering with Euclidean distance for both genes and arrays as a similarity metric. Average linkage was used as a clustering method. Gene ontology analysis was performed using ‘findGO.pl’ program in HOMER 4.7 software. For analyzing distal regulatory elements near differentially regulated genes in mice, ENCODE ChIP-Seq data for H3K27ac and DNAse hypersensitive sites for mouse liver were used. Mice RNA-Seq data have been submitted to NCBI Gene Expression Omnibus under accession number GSE82106. A summary of the NGS samples used in the analysis and lists of genes in heatmaps are found in Supplemental Files: NGS experiments A–E.

Available global run-on sequencing (GRO-seq) data from (1) normoxic and hypoxic HUVECs (GEO: GSE94872) and (2) pig heart ischemia samples (GEO: GSE81155) were used for analysis. Single-cell sequencing data from mouse tissues are publicly available at tabula muris.ds.czbiohub.org/ [27].

### Immunohistochemistry

Paraffin embedded tissue samples were sectioned (4–10 µm), stained with primary antibodies CD31, VEGF-A, BMP2 and TAZ (please see the Major Resources Table in the Supplemental Material for details) and counterstained with Harris Hematoxylin. Imaging was performed using LSM700 Zeiss confocal microscope or Eclipse Ni-E Nikon microscope (10 ×/0.3 or 20 ×/0.5 Plan Fluor objectives, 4908 × 3264 frame size). In confocal microscope, 405/488/555 nm diode lasers were used together with the appropriate emission filters (10 ×/0.3 or 20 ×/0.5 PlanApo objectives, 512 × 512 and 1024 × 1024 frame sizes). Image processing was performed by ImageJ [28] and quantitative analysis by NIS-Elements (*n* = 4–6 images/tissue/animal).

### Enzyme-linked immunosorbent assay

Plasma and liver VEGF concentrations were measured using ELISA for human VEGF (R&D Systems) according to manufacturer’s instructions.

### Angiogenesis bead assay

A 3D in vitro model mimicking angiogenesis was performed as described previously [29]. Shortly, HUVECs (p3) were seeded on top of collagen-coated Cytodex 3 beads (GE Healthcare, Little Chalfont, UK) and cultured in a fibrin gel (fibrinogen, aprotinin, thrombin; Merck KGaA, Darmstadt, Germany). HPF cells were cultured on top of the fibrin gel. Cell culture was maintained with EBM media with additives (Lonza, Basel, Switzerland), and stimulated with VEGF and/or BMP2/4/6 recombinant proteins. Media and protein stimulations (100 ng/ml) were replaced every other day during the 3–7 days follow up. After fixation, the cells were labeled with Phalloidin-A635 (Thermo Fisher Scientific), VEGFR2 antibody (Cell Signaling Technology, Danvers, MA), and DAPI. Images were taken with confocal laser scanning microscope (Zeiss LSM800). 405/555 nm diode lasers were used together with the appropriate emission filters (10 ×/0.3 PlanApo objective, 1024 × 1024 frame size). Image processing and analysis was performed by ImageJ [28] or Angiosys softwares (Cellworks, Caltag Medsystems Ltd., Buckingham, UK). The angiogenic sprout analysis was performed from endothelial sprouts containing > 1 nuclei (29–43 beads/group). Segmented area of endothelial cells was detected by Angiosys.

### In vitro tube formation assay

HUVECs were seeded on 6-well plates and transfected with 5 or 10 nM siRNAs. After 24 h, cells were detached and transferred to growth factor reduced matrigel (Corning, Inc., New York, NY) coated 48-well plates (40,000 or 50,000 cells/well). Cells were imaged with IncuCyte® S3 Live-Cell Analysis System (Sartorius, Göttingen, Germany) or Olympus IX71 microscope (Tokyo, Japan) using a × 4 objective lens. After 16 h, the cells were fixed with 1% glutaraldehyde–2% PFA solution. Tube formation was analyzed with ImageJ Angiogenesis Analyzer.

### CyQUANT cell proliferation assay

HUVECs were seeded on 96-well plates at 4000 cells/well and transfected with 10 nM siRNAs for 48 h. Detection of cellular DNA by CyQUANT cell proliferation assay was performed according to manufacturer’s instructions using absorbance of 530 nm (Thermo Fisher Scientific).

### Western blot

Confluent cultures of HUVECs were washed with PBS, followed by starvation of cells for 16 h with EGM medium supplemented with 0.5% FBS. For whole cell protein extraction cells were either transfected with 5 nM siRNAs (siCTRL, siBMP2, siBMP6 or siTAZ) or treated with BMP6 (100 ng/ml; 7 h, 10 h, 14 h, 24 h) and after lysed with Tris–HCl buffer (50 nM Tris–HCl, 150 nM NaCl, 1 mM EDTA, 1% Triton X-100, Na-deoxycholate, 0.1% SDS, 10% glycerol). For cell compartment fragmentation HUVECs treated with BMP2 or BMP6 (100 ng/ml; 1 h, 2 h) were harvested, and cytoplasmic and nuclear proteins were extracted with NE-PER kit (Thermo Fisher Scientific) according to manufacturer’s instructions. Protease and phosphatase inhibitors (Roche, Basel, Switzerland) were added to lysing reagents. After determining the protein concentrations with Pierce™ BCA kit (Thermo Fisher Scientific) equal amounts of proteins were loaded on the gel from each sample (5 or 10 µg). Primary antibodies: YAP/TAZ, phospho-TAZ, VEGFR2, histone H3, β-actin (Cell Signaling Technology) were used. Horse radish peroxidase (HRP) conjugates were used as secondary antibodies. Detection of antigen–antibody complexes was performed with Pierce™ ECL western blotting substrate (Thermo Fisher Scientific) and ChemiDoc™ MP Imaging System (Bio-Rad, Hercules, CA). Quantitative analysis of adjusted volume intensities of the protein bands from immunoblots was performed by ImageLab 6.0 software. Intensity values were normalized to adjusted volume intensities of both loading control and untreated sample.

### Statistical analysis

Statistical analyses were performed with GraphPad Prism software (San Diego, CA). Mann–Whitney *U*-test, One-way ANOVA followed by Dunnett’s multiple comparison test (*-indication) or Unpaired *t*-test (#-indication) were used. *Ρ* < 0.05 was used to define statistical significance.

### Ethics statement

Animal experiments were approved by National Experimental Animal Board of Finland and carried out in accordance with guidelines of the Finnish Act on Animal Experimentation. Collection of umbilical cords for cell isolation was approved by Ethics Committee of the Kuopio University Hospital (Kuopio, Finland, 341/2015).

## Results

### VEGF induces expression of bone morphogenetic proteins in vivo

Although the function of VEGF has been studied in various cell models, transcriptional programming that leads to angiogenesis in vivo, and the role of other growth factors regulating this event have remained elusive. To study this, we did systemic adenovirus vector mediated VEGF gene transfer in C57/Bl6 mice and performed RNA-sequencing (RNA-Seq) to detect all steady state mRNAs. AdCMV, a vector with only the promoter region and no transgene, was used as a control.

**Fig. 1:**
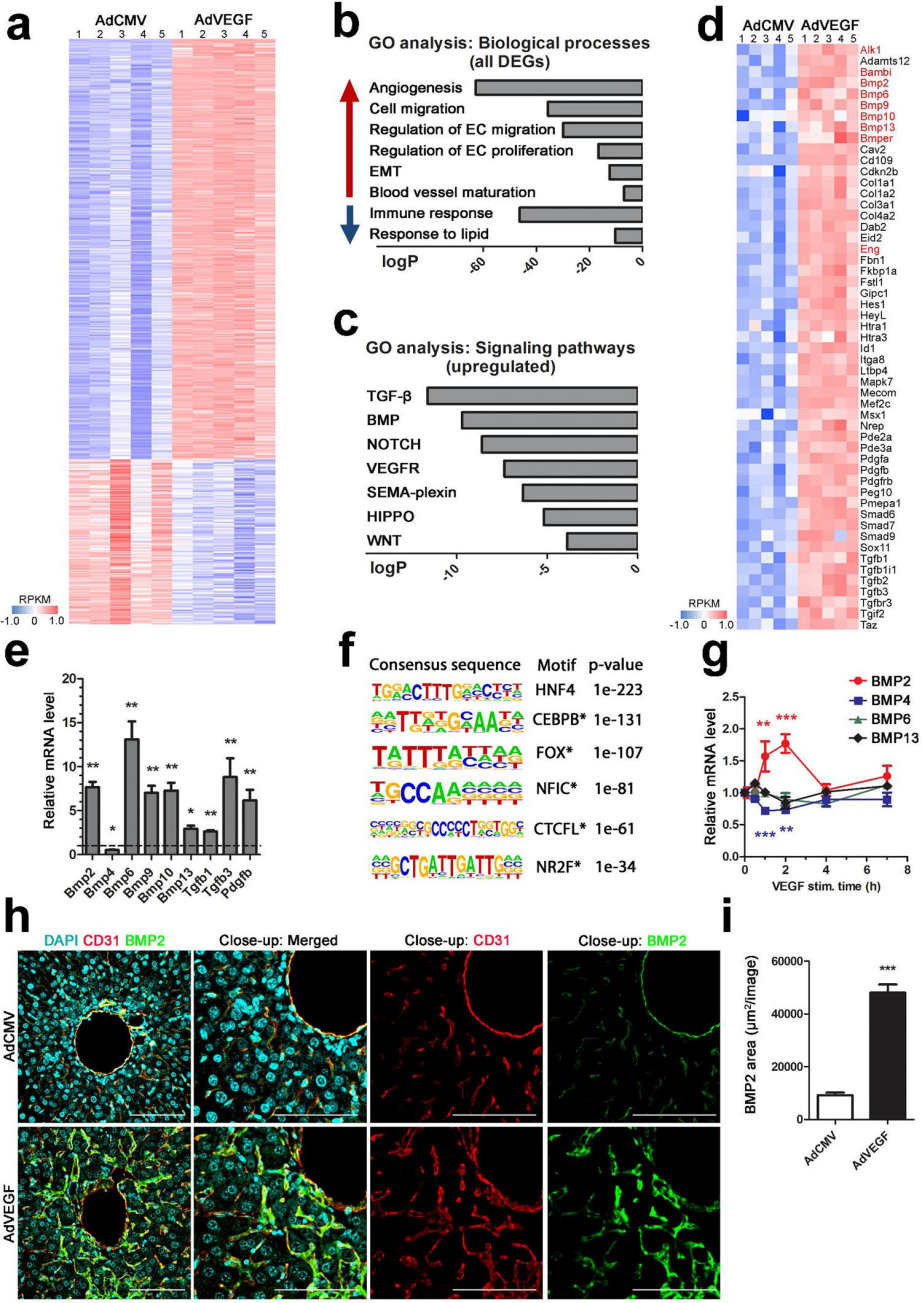
VEGF induces expression of various growth factors in vivo including bone morphogenetic proteins. **a**–**f** RNA-sequencing data from liver of C57/Bl6 mice after VEGF gene transfer. AdCMV was used as a control vector containing only the promoter region and no transgene (i.v., 6 days, *n* = 5/group). **a** Among differentially expressed genes between transgene and control vector treated mice livers, 1284 genes were upregulated (red) and 504 downregulated (blue) in the AdVEGF group. Heatmap of normalized, log transformed and centered RPKM gene expression values are shown. **b** Gene ontology (GO) analysis revealed enrichment of biological processes such as angiogenesis, endothelial cell (EC) migration and blood vessel maturation among the upregulated genes (red arrow). Immune response and lipid metabolism related processes were among the downregulated genes (blue arrow; log*P* < − 6). **c** Cell signaling pathways, such as TGFβ, BMP, NOTCH and VEGFR were enriched in GO analysis among the upregulated genes (log*P* < − 3). **d** Heatmap of genes related to TGFβ- and BMP-signaling pathways after AdVEGF gene transfer, including Bmps, BMP-binding endothelial regulator (Bmper), BMP and activin membrane-bound inhibitor (Bambi), and endoglin (Eng). Heatmap of normalized, log transformed and centered RPKM gene expression values are shown. BMP receptors and ligands are marked in red. **e** Sequencing results were validated with RT-qPCR showing mRNA expression levels of Bmps, TGFβ 1/3 and Pdgf-β (*n* = 3–5 animals/group). **f** Consensus sequences of the enriched motifs predicted to function as activated transcription factors in VEGF-treated mice liver are presented. Enriched motifs were associated with angiogenesis, or BMP signaling (marked with asterisk). **g** VEGF stimulation (50 ng/ml) was shown to induce BMP2 (red) mRNA expression and downregulate BMP4 (blue) expression in primary endothelial cells (HUVEC) detected by RT-qPCR. No significant changes were detected in mRNA levels of BMP6 (green) or BMP13 (black). **h** A representative image of BMP2 protein expression and localization to CD31-stained liver sinusoidal endothelial cells after VEGF gene transfer detected by confocal microscopy (× 20 magnification, close-up × 25; red, CD31; green, BMP2; blue, DAPI-labeled nuclei, scale bars 100 µm). **i** Quantification of BMP2-positive area showed significant upregulation of the protein in VEGF-treated mice in comparison to control mice (*n* = 5 animals/group, 30–31 images/group). For all RT-qPCR experiments, mean ± SEM are presented, 2–3 independent experiments were performed in triplicates. *Ρ*-values < *0.05, <  **0.01, ***< 0.001

Successful gene transfer was first determined by detecting increased VEGF mRNA and protein levels in liver and plasma at 6 days after the gene transfer (Supplementary Fig. 1a, b, d). Additionally, VEGF-induced vessel dilatation was demonstrated in both the centrilobular and periportal areas of the liver (Supplementary Fig. 1c, e). By RNA-Seq, differential gene expression patterns were detected in VEGF-treated mice, leading to identification of 1284 upregulated and 504 downregulated genes in comparison to control group (Fig. 1a). Gene ontology analysis revealed that differentially expressed genes were involved in cellular processes, such as angiogenesis, endothelial cell migration and proliferation (Fig. 1b). Particularly, transforming growth factor (TGFβ)-, BMP-, NOTCH- and VEGF-receptor (VEGFR) signaling pathways were enriched in VEGF-treated mice (Fig. 1c). Altogether, 53 genes were related in TGFβ- or BMP-signaling pathways (Fig. 1d). For example, BMPs 2, 6, 9, 10 and 13 and their regulators BMP-binding endothelial regulator (Bmper), Bambi and endoglin (Eng) were upregulated. RNA-Seq results were validated by RT-qPCR, showing significant difference in regulation of Bmps, Tgfβ 1/3 and platelet-derived growth factor Pdgf-β between VEGF-treated mice and the control group (Fig. 1e).

To detect possible regulatory transcription factor-binding sites in VEGF-treated mice, de novo motif analysis was additionally performed from differentially regulated genes. Intergenic regions located 3 kB away from the transcription start site (TSS) and 10 kB from the TTS of any known RefSeq or UCSC gene were analyzed. HNF4, CEBPB, FOX class, NFIC, CTCFL and NR2F were identified as potential transcription factors implicated in transcriptional response to VEGF stimulus (Fig. 1f). All the identified factors have been previously connected to angiogenesis or BMP signaling [30–36].

Next, to gain insight into cell type specific expression levels of BMPs and their receptors in mouse liver, publicly available single-cell sequencing database Tabula Muris were used. BMP2 and BMP6 were found to be expressed in endothelial cells and hepatocytes (Supplementary Fig. 2a, c), together with their receptors ALK2, ALK3 and BMPR2 (Supplementary Fig. 2e). Instead, only a very low expression levels (BMP4/5/9), or no expression at all were found with other BMPs in endothelial cells (BMP1, BMP3, BMP7, BMP8, BMPs 10–15). To define the role of VEGF in mediating expression of BMPs, VEGF stimulation experiment was further performed in human primary endothelial cells. By RT-qPCR, BMP2 mRNA was shown to be significantly upregulated and BMP4 downregulated after VEGF treatment (Fig. 1g). No change was observed with other BMPs (BMP6, BMP9, BMP10 or BMP13). Accordingly, upregulation and localization of BMP2 protein to liver sinusoidal endothelial cells was confirmed by immunohistochemistry and quantitative analysis in VEGF-treated mice in areas with high endothelial cell proliferation and sprouting (Fig. 1h, i).

Altogether, our omics approach demonstrates that VEGF induces a pleiotropic effect in vivo and regulates expression of multiple growth factors, including members of the TGFβ superfamily. BMP2 or BMP4 have not been previously linked to VEGF-induced effects in vivo, although they have shown to mediate angiogenesis [10, 17, 19]. This is the first time VEGF-mediated long-term effects after gene transfer have been detected by next-generation sequencing (NGS) in mice.

### BMPs are regulated in ischemia and hypoxic endothelial cells

**Fig. 2:**
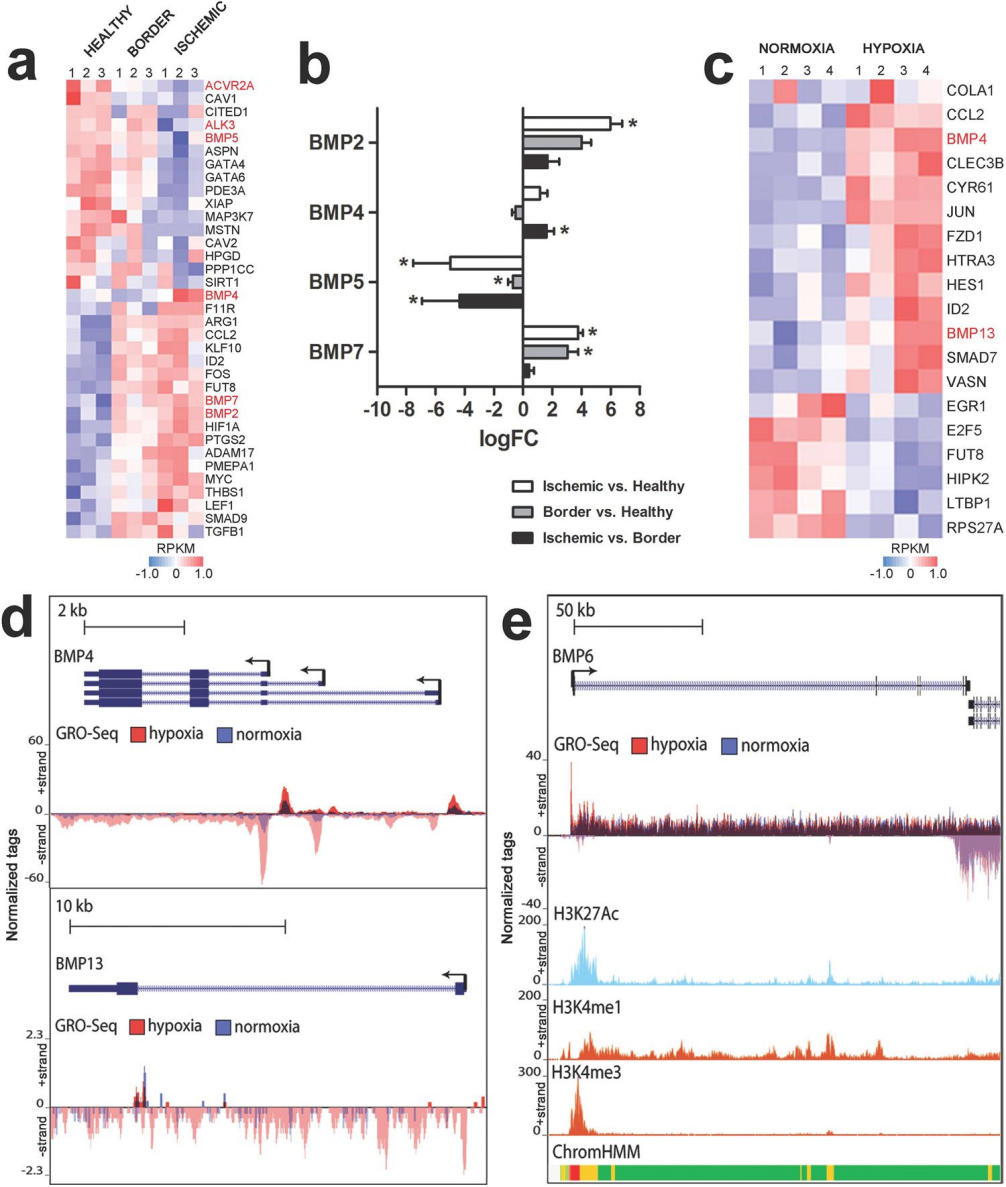
BMPs are regulated after acute myocardial ischemia in pigs and in hypoxic endothelial cells. **a** Global run on -sequencing (GRO-Seq) data revealed that genes involved in TGFβ- or BMP-signaling pathways were differentially expressed between healthy-, border- and ischemic-zones of a pig myocardium after acute infarction. BMP ligands and receptors are marked in red. Heatmap of normalized, log transformed and centered RPKM gene expression values are shown (red, upregulated genes; blue, downregulated genes; *n* = 3/zone). **b** RPKM value derived log fold changes of BMP2/4/5/7 mRNA expression levels detected by GRO-Seq in healthy-, border- and ischemic-zones of a pig myocardium. Significant differences in gene expression levels are marked with an asterisk (*n* = 3/zone, mean ± SEM, FDR-value < 0.05*). **c** Heatmap of hypoxia induced genes related to TGFβ- or BMP-signaling pathways in HUVECs in comparison to normoxia treated cells. BMP ligands are marked in red. Normalized, log transformed and centered RPKM gene expression values are shown (GRO-Seq, *n* = 4/group; red, upregulated genes; blue, downregulated genes). **d** UCSC Genome browser image depicting normalized GRO-Seq tag counts of BMP4 and BMP13 genes in normoxia (blue) and hypoxia (red). Hypoxia-induced transcription of BMP4 and -13 in HUVECs is shown. **e** UCSC Genome browser image depicting normalized GRO-Seq tags in hypoxic or normoxic HUVECs. In ChIP-Seq data H3K27Ac tags show enhancer regions (light blue), and H3K4me3 nascent RNA in promoter regions (orange). BMP6 exhibits a transcriptionally paused phenotype. Chromatin state segmentation is shown (ChromHMM). Active promoter region (red), strong enhancer (orange), transcriptional transition/elongation (green)

Previously, hypoxia has been shown to induce VEGF upregulation and neovessel formation [37]. To understand the role of BMP family members in hypoxia-induced angiogenesis and to compare it with VEGF-transgene induced effects, we next used our recent datasets measuring nascent RNA transcription by global run-on sequencing (GRO-Seq) [26, 38]. Nascent RNAs were sequenced from three regions of the porcine heart after acute ischemia: ischemic, border zone and healthy myocardium (GSE81155). Acute ischemia was induced by cardiac catheterization, and samples were collected after 24 h. We found 35 genes from TGFβ- and BMP-signaling pathways that were regulated differentially among the 3 regions, from which pro-angiogenic BMPs 2/4/7, as well as BMP-signaling regulators SMAD9 and LEF1 were upregulated, and BMP5 downregulated in the ischemic areas (Fig. 2a, b).

Single-cell RNA-sequencing data obtained from Tabula Muris further confirmed that several cell types in the heart, including endothelial cells, fibroblasts, smooth muscle cells and cardiomyocytes express BMPs and their receptors (Supplementary Fig. 2b, d, f). BMP2 was particularly expressed in smooth muscle cells, whereas BMP4 was expressed in both endothelial cells and fibroblasts (Supplementary Fig. 2d). To further identify hypoxia-induced effects specifically in endothelial cells, expression of BMPs was investigated from normoxic and hypoxic HUVECs (8 h, 1% of O_2_; GSE94872) [26]. BMP 2, 4, 6 and 13 were shown to be highly transcribed in normoxia, whereas very low transcription levels were detected with other BMP family members. Nineteen genes involved in TGFβ- and BMP-signaling pathways showed differential nascent-RNA production in hypoxic HUVECs, in comparison to normoxic cells (GRO-Seq, Fig. 2c), including BMP4 and BMP13 (Fig. 2d). Further characterization of the transcriptional profiles revealed that BMP6 exhibited a transcriptionally paused phenotype in hypoxic endothelial cells with significantly higher peak of nascent RNA produced from the promoter region (H3K4me3; Fig. 2e). A release of RNA polymerase from the promoter region to productive elongation thus likely limits BMP6 production in hypoxia.

Taken together, besides being regulated in VEGF-induced angiogenesis and in normoxic endothelial cells, BMP family members are regulated in ischemia and in hypoxic endothelial cells. This implicates that BMPs are important modulators of angiogenesis that could crosstalk with VEGFR2 signaling pathway. As only BMP4 was shown to be induced in hypoxic endothelial cells, various hypoxia-related genes may regulate BMP signaling and expression in other cell types of the ischemic tissue.

### VEGF-mediated vessel sprouting is regulated by BMP2 and BMP6

**Fig. 3:**
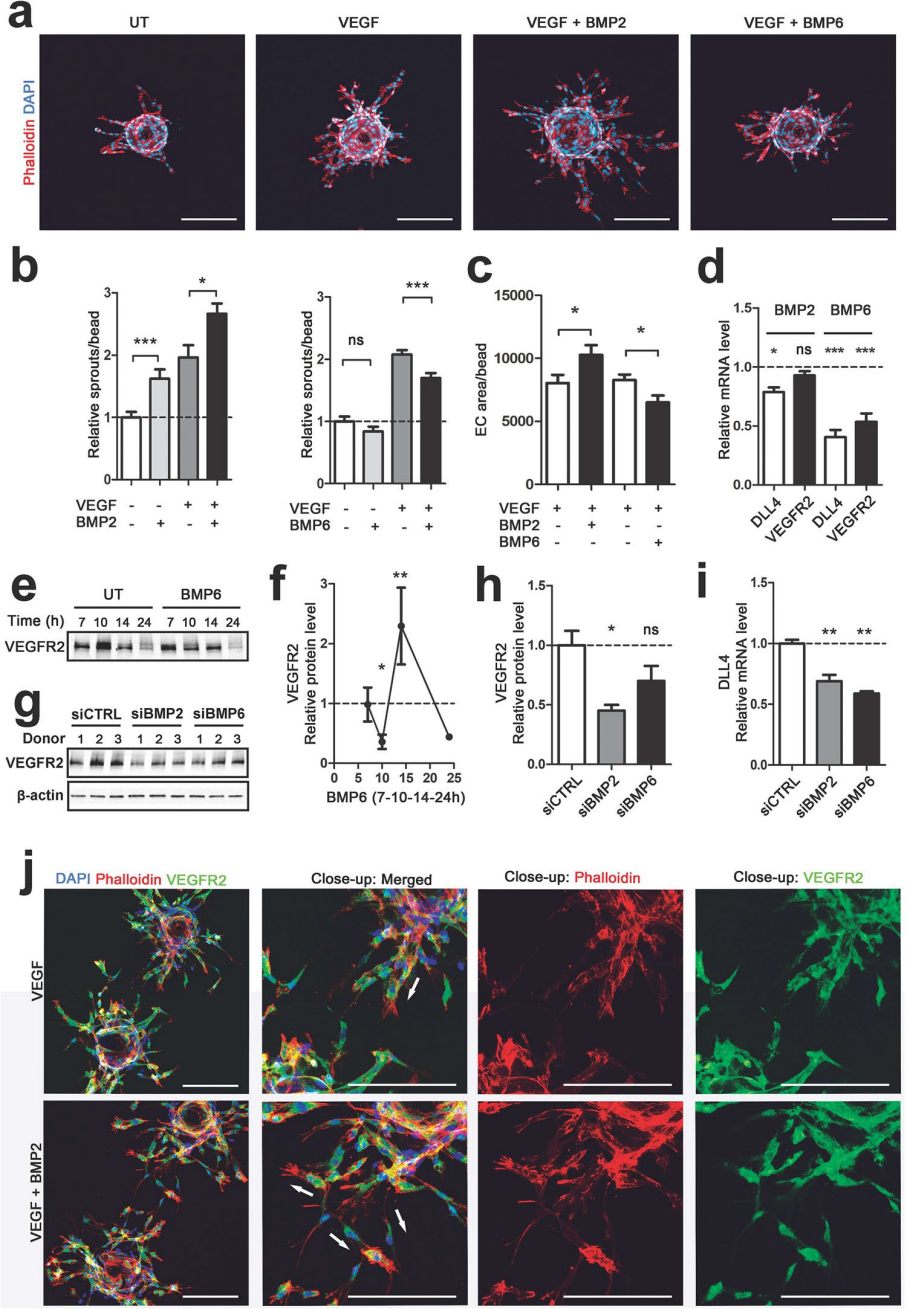
BMPs modulate VEGF-induced angiogenesis and regulate tip cell associated markers VEGFR2 and DLL4. **a**–**c**, **j** Fibrin bead assay enables modeling of angiogenesis in long time points in vitro. HUVECs were grown on collagen-coated beads and embedded in a fibrin gel. HPF cells were put on top of the fibrin gel to enable lumen formation. BMP/VEGF stimulations were performed every other day. At d7 cells were fixed, and F-actin was labeled with phalloidin-A635 (red) and nuclei with DAPI (blue). Imaging was performed by confocal microscopy. **a** At 7-day timepoint co-stimulations of BMP2 or BMP6 with VEGF were shown to regulate endothelial cell sprouting in comparison to untreated (UT) or VEGF-treated cells. BMP2 was shown to be pro-angiogenic and BMP6 anti-angiogenic with VEGF (red, phalloidin-labeled F-actin; blue, DAPI-labeled nuclei, scale bars 200 µm). **b** BMP2 increased the amount of endothelial cell sprout per bead both alone and in co-stimulation with VEGF. BMP6 decreased VEGF-induced sprouting, whereas BMP6 stimulation alone did not have an effect. ImageJ analysis, *n* = 30–33 beads/group, sprouts included in the analysis contained ≥ 1 DAPI-labeled nuclei. **c** Similar effects of BMP2 (pro-angiogenic) and BMP6 (anti-angiogenic) on VEGF-induced endothelial cell sprouting were detected by analyzing the endothelial cell area per bead. Angiosys analysis, *n* = 29–43 beads/group, sprouts included in the analysis contained ≥ 1 DAPI-labeled nuclei. **d** BMP6 stimulation (100 ng/ml, 7 h) decreased tip cell markers DLL4 and VEGFR2 mRNA expression in HUVECs. BMP2 led to slight downregulation of DLL4 but did not affect VEGFR2. RT-qPCR detection. **e** and **f** BMP6 stimulation (100 ng/ml) led to fluctuation in the protein amount of VEGFR2 in HUVECs. Western blot was performed from 7, 10, 14 and 24 h timepoints after BMP6 stimulation. **e** A representative image of VEGFR2 protein levels in BMP6 stimulated HUVECs. Pooled samples from 2 to 3 donors, *n* = 3/donor. **f** VEGFR2 protein levels after BMP6 stimulations were analyzed from 2 to 3 donors/treatment, *n* = 3/donor, normalized to β-actin and untreated samples. **g** and **h** Inhibition of endogenous BMP2 and BMP6 with specific siRNAs (siBMP2 or siBMP6, 48 h transfection, 5 nM) led to a decrease in VEGFR2 protein amount in HUVECs. Western blot analysis, 3 donors/treatment, *n* = 3/donor (pooled in the blot), beta-actin was used to normalize target protein levels. **i** siRNA treatments (48 h transfection, 5 nM) targeting to BMP2 and BMP6 caused downregulation of DLL4 mRNA. RT-qPCR detection. **j** Representative images of the sprout front between two beads are presented. 3 days of co-stimulation of BMP2 and VEGF (both 100 ng/ml), induced the growth of narrow endothelial cell sprouts in various orientations. With VEGF stimulation alone, the endothelial tubes were wider and had an organized sprout front (× 10 magnification, close-up × 15; green, VEGFR2; red, phalloidin-labeled F-actin; blue, DAPI-labeled nuclei, scale bars 200 µm). White arrows indicate the sprout orientation. In all images, mean ± SEM are presented and 2–3 independent experiments were performed in triplicates. *Ρ*-values < *0.05, < **0.01, < ***0.001

Due to their pro-angiogenic characteristics [10, 17, 19], and our findings in normoxic and hypoxic endothelial cells, BMPs 2, 4 and 6 were selected for further studies. First, a co-culture angiogenesis bead assay was performed, containing both primary human endothelial cells and fibroblasts. This assay enables a long-term follow-up of endothelial sprouting, sprout elongation and lumen formation in vitro [29]. After 7 days in culture, a stimulation with BMP2, and a co-stimulation of BMP2 and VEGF proteins, were both shown to induce endothelial sprouting (Fig. 3a; Supplementary Fig. 3a, b). By quantitative image analysis, a significant increase in the number of endothelial sprouts and cell area were observed in the BMP2 and VEGF co-stimulation group as compared to the VEGF group (Fig. 3b, c). At d3 with solely VEGF, the endothelial sprouts were wide and oriented towards each other from the closely located beads. The co-stimulation of VEGF and BMP2 instead induced formation of narrower sprouts with tip cells migrating to various orientations (Fig. 3j).

Conversely to BMP2, a co-stimulation of VEGF and BMP6 led to a decreased endothelial sprout formation and cell area in the co-culture angiogenesis bead assay (Fig. 3a–c). No differences were observed with BMP4 or BMP6 alone (Supplementary Fig. 3a, b), or in co-stimulation experiments with BMP4 and VEGF (Supplementary Fig. 3b).

Next, to investigate the mechanisms behind BMP6 and BMP2 induced effects on VEGF-mediated endothelial sprouting, expression of tip cell markers was detected from BMP-stimulated or siRNA treated HUVECs by RT-qPCR and western blot. At 7 h after protein stimulation, BMP6 was shown to decrease mRNA expression of both VEGFR2 and Delta Like Canonical Notch Ligand 4 (DLL4; Fig. 3d), thus explaining its function as an anti-angiogenic factor in the co-culture angiogenesis bead assay. No change was observed in the expression levels of VEGFR1 or VEGF co-receptors neuropilin 1 and 2 (NRP1 and NRP2; Supplementary Fig. 3c). Accordingly, BMP6 stimulation led to significant changes in VEGFR2 protein levels detected by western blot (Fig. 3e, f). BMP2 protein stimulation instead did not have any effects on the mRNA expression levels of VEGFRs, NRPs or VEGF-A (Fig. 3d; Supplementary Fig. 3c). However, expression of DLL4 was shown to be reduced (Fig. 3d). Silencing of BMP2 or BMP6 with siRNAs further confirmed that DLL4 and/or VEGFR2 are regulated by BMPs (Fig. 3g–i).

To conclude, this is the first time that a co-culture angiogenesis cell model containing both endothelial cells and fibroblasts has been used to evaluate effects of BMPs on VEGF-induced endothelial sprout formation. Our data demonstrate that BMP2 and BMP6 act as regulators of VEGF signaling by differentially modifying the availability of VEGFR2 and regulating the expression of the Notch ligand DLL4. As reduced level of DLL4 has been linked to induction of poorly matured vessels and defective cell fate specification [39, 40], this or possible differences in activation of RhoGTPases by BMP2 and VEGF leading to re-organization of actin cytoskeleton [41–43] could explain the observed morphological differences induced by BMP2 in VEGF-mediated endothelial sprouting.

### BMP6 induces nuclear localization of TAZ which regulates VEGFR2

TGFβ and VEGF have been previously linked to Hippo signaling pathway [44, 45] that is known to regulate important cell functions in angiogenesis, such as cell proliferation [46] and cell survival [47]. Hippo signaling also acts as an integrator of various signaling pathways participating in angiogenesis, including Wnt, GPCR, EGF and Notch [45]. To determine if VEGF-induced endothelial sprouting is regulated via BMP2 and BMP6 mediated Hippo signaling, we next analyzed the cellular localization and expression of Hippo pathway components.

**Fig. 4:**
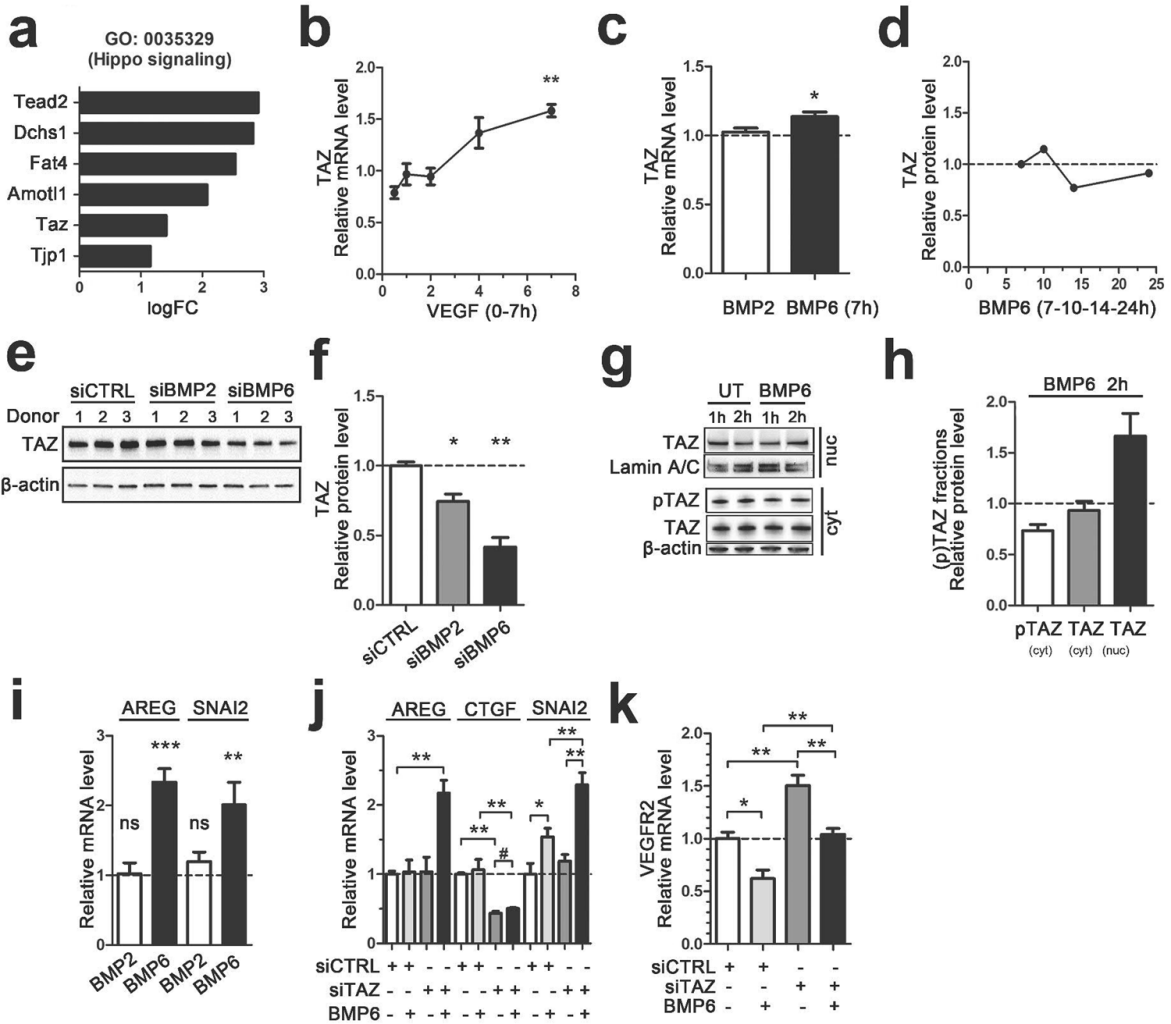
BMP6 relocalizes TAZ and upregulates Hippo target genes in endothelial cells. **a** Hippo signaling pathway genes detected in C57/Bl6 mice after VEGF gene transfer by RNA-Seq, including transcriptional activity mediator Taz and Tead2. LogFC values are shown (i.v.; 6 days; *n* = 5/group). **b** VEGF (50 ng/ml, 7 h) stimulation increased TAZ mRNA expression level in HUVECs. RT-qPCR detection. **c** BMP6 but not BMP2 stimulation (100 ng/ml, 7 h) upregulated TAZ mRNA expression in HUVECs. RT-qPCR detection. **d** BMP6 stimulation (100 ng/ml) did not affect TAZ protein amount in 7, 10, 14 or 24 h timepoints in HUVECs. Western blot detection of TAZ was performed from pooled samples of 2–3 donors (*n* = 3/donor) and normalized to beta-actin and untreated samples. **e** and **f** Inhibition of endogenous BMP2 and BMP6 with specific siRNAs (siBMP2 or siBMP6, 48 h transfection, 5 nM) led to a decrease in TAZ protein amount in HUVECs in comparison to control group (siCTRL). Western blot analysis, 3 donors/treatment, *n* = 3/donor (pooled in the blot), beta-actin was used to normalize target protein levels. **g** and **h** TAZ protein expression in cytoplasmic and nuclear fractions of untreated (UT) and BMP6 (100 ng/ml; 1 h, 2 h) stimulated HUVECs. **g** A representative image of nuclear and cytoplasmic TAZ, cytoplasmic phosphorylated TAZ (pTAZ) and loading controls Lamin a/c (nucleus), β-actin (cytoplasm). **h** 2 h BMP6 stimulation increased nuclear (nuc) TAZ whereas decreased cytoplasmic (cyt) pTAZ and TAZ. Western blot analysis with normalization to Lamin A/C (nucleus) or β-actin (cytoplasm) and untreated samples. **i** BMP6 but not BMP2 stimulations (100 ng/ml, 7 h) increased Hippo signaling targets Amphiregulin (AREG) and Snail Family Transcriptional Repressor 2 (SNAI2) mRNA expression in HUVECs. RT-qPCR detection. **j** BMP6 stimulation (100 ng/ml, 7 h) on siTAZ treated (48 h transfection, 10 nM) HUVECs led to upregulation of mRNA expression of Hippo signaling target genes AREG, CTGF and SNAI2 in comparison to siTAZ cells without BMP6 stimulation. RT-qPCR detection, statistical tests Mann–Whitney (*) or unpaired (#) *t*-test. **k** VEGFR2 mRNA expression was upregulated in siTAZ treated (48 h transfection, 10 nM) and BMP6 stimulated (100 ng/ml, 7 h) HUVECs in comparison to control group (siCTRL with BMP6 stimulation). RT-qPCR detection. In all images, mean ± SEM are presented. 2–3 Independent experiments were performed in triplicates. *Ρ*-values < *0.05, < **0.01, < ***0.001

Transcriptional activity mediators of Hippo signaling, TAZ (transcriptional co-activator with PDZ-binding domain; also known as WW domain containing transcription regulator 1, Wwtr1) and TEA domain transcription factor 2 (TEAD2) were shown to be upregulated in VEGF-treated mice detected by RNA-Seq and RT-qPCR (Fig. 4a; Supplementary Fig. 4e). Accordingly, in functional assays, silencing of TAZ or TEAD2 by siRNAs led to significantly reduced endothelial cell proliferation, and/or tube formation (Supplementary Fig. 4a, b), emphasizing the importance of Hippo signaling in regulating endothelial cell function. Upregulation and localization of Taz protein to liver sinusoidal endothelial cells were further confirmed by immunohistochemistry in VEGF-treated mice (Supplementary Fig. 4d). In experiments with endothelial cells, VEGF or BMP6 stimulations induced modest TAZ mRNA and/or protein upregulation, whereas no effect was observed with BMP2 (Fig. 4b–d). siRNA silencing experiments confirmed that particularly BMP6 regulates expression of TAZ at the protein level detected by western blot (Fig. 4e, f).

Besides transcriptional and translational regulation, TAZ is regulated via shuttling of the protein between nucleus and cytoplasm [45], leading to expression of the Hippo target genes. Although VEGF is known to induce nuclear localization of Hippo pathway components [46, 48], a similar role of BMP2 and BMP6 is not known. To test this, we stimulated primary endothelial cells with BMP proteins, and analyzed TAZ expression in the cytoplasmic and nuclear fractions by western blot. At 2 h time point, increased nuclear fraction of TAZ protein was detected in BMP6 stimulated cells (Fig. 4g, h), identifying BMP6 as the first BMP family member to be associated with TAZ-Hippo pathway regulating shuttling of TAZ to nucleus. 2 h BMP2 stimulation instead did not activate TAZ nuclear translocation (Supplementary Fig. 4f, g). Further RT-qPCR assays were performed to detect known Hippo signaling target genes after BMP stimulation: amphiregulin (AREG), Snail Family Transcriptional Repressor 2 (SNAI2) and connective tissue growth factor (CTGF) [49–51]. 7 h BMP6 stimulation induced mRNA expression of both AREG and SNAI2 whereas BMP2 stimulation had no effect (Fig. 4i). Concomitantly in siTAZ treated cells, BMP6 stimulation upregulated AREG, CTGF and SNAI2 (Fig. 4j). Cysteine Rich Angiogenic Inducer 61 (CYR61) linked with Hippo pathway [52] was not altered after BMP2 or BMP6 stimulus (Supplementary Fig. 4h, i).

Finally, to understand whether TAZ regulates BMP6-mediated downregulation of VEGFR2 and thus reduces VEGF-induced angiogenic effects, VEGFR2 expression level was determined from BMP6 stimulated and siTAZ treated cells. Significant increase in VEGFR2 mRNA level was detected with BMP6 stimulated, siTAZ treated cells in comparison to control cells (Fig. 4k), thus demonstrating that TAZ is a regulator of BMP6-mediated VEGFR2 expression.

### BMP6 mediates neovessel formation

**Fig. 5:**
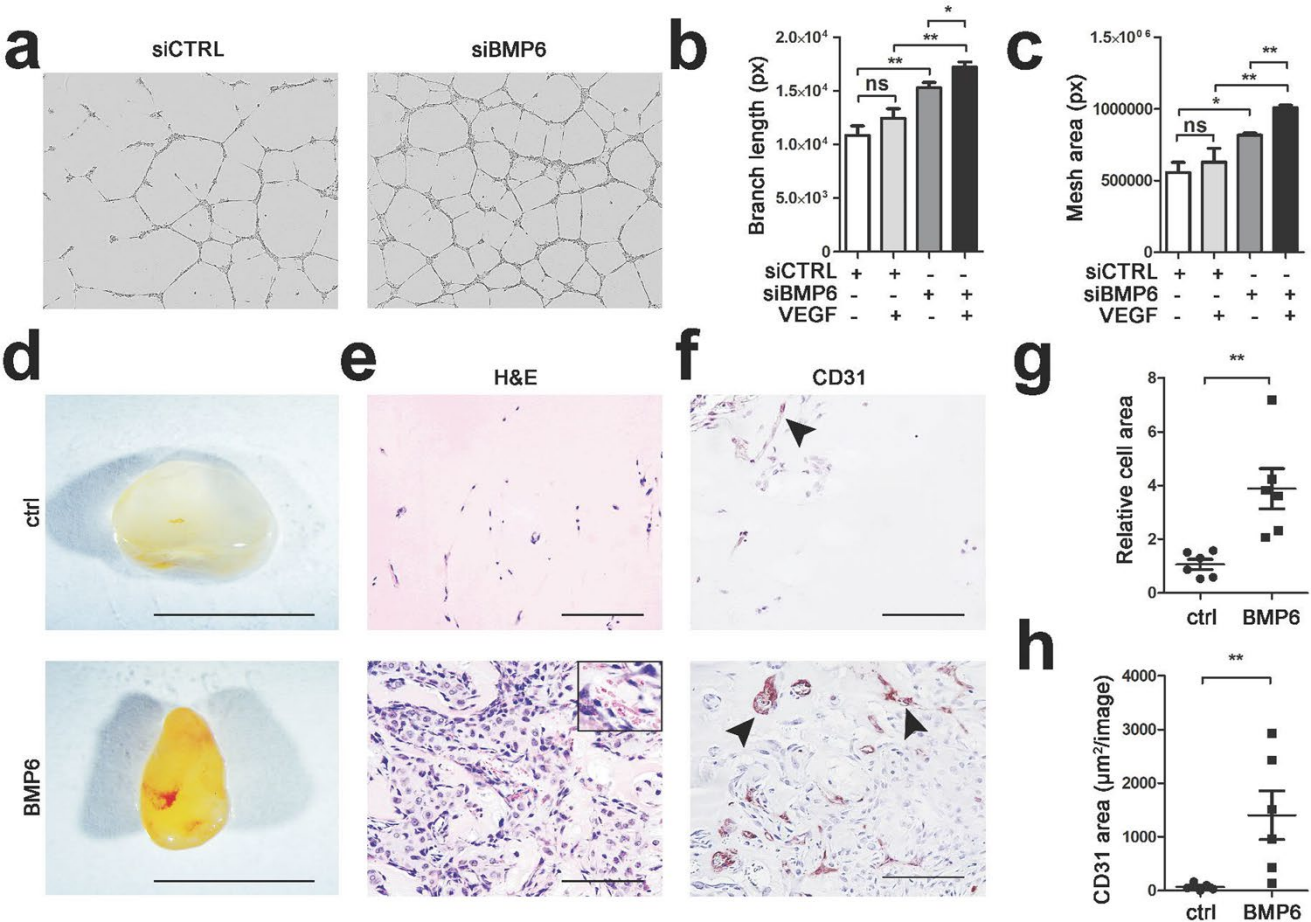
BMP6 induces neovessel formation in vivo. **a–c** Matrigel tube formation assay (16 h) was performed with BMP6 siRNA in HUVECs (7 h VEGF stimulation, 100 ng/ml). **a** Representative images of siCTRL and siBMP6 treated cells. Imaging was performed with IncuCyte® S3 Live-Cell Analysis System (magnification × 4). **b** Quantitative analysis of total length of endothelial branch network. **c** Quantitative analysis of total endothelial mesh area. In all data, mean ± SEM are presented and two independent experiments were performed in triplicates. *Ρ*-values < *0.05, < **0.01, < ***0.001. **d–h** Angiogenic effect of BMP6 was evaluated with matrigel plug assay in nude mice. Growth factor reduced matrigel with S1P supplementation alone (negative control) or together with BMP6 were injected s.c. into nude female mice (6 weeks old, *n* = 6 mice/treatment). **d** Representative images of matrigel plugs after resection at d7. Plugs with BMP6 recombinant protein were thoroughly reddish in color and had denser structure in comparison to S1P control. Scale bars 1000 µm. **e** Representative images of H&E-stained 10 µm paraffin sections of the matrigel plugs showing the cell infiltration induced by BMP6. Red blood cells were observed in BMP6 plugs indicating blood circulation within the plug (close-up). Scale bars 100 µm. **f** Representative images of CD31-labeled paraffin sections of the matrigel plugs showing endothelial cells (CD31-label, arrow heads). Scale bars 100 µm. **g** Quantitation from H&E-stained paraffin sections showed a significant increase in cell area in plugs with BMP6 in comparison to control plugs. **h** Quantitation of CD31-positive area showed a significant increase in the vascular area in plugs with BMP6 in comparison to control plugs. Quantitation was performed with NIS-Elements Analysis Software. Mean ± SEM are presented (4–8 images/plug, *n* = 5–6 plugs/condition). For simplicity, S1P-negative controls (ctrl) of each mouse are combined in the graph (*n* = 15). *Ρ*-values < *0.05, < **0.01, < ***0.001

Effect of BMP6 to angiogenesis was further tested in endothelial cell tube formation assay and in nude mice by matrigel plug assay. Silencing of BMP6 by siRNA resulted in an increase of both total length of endothelial cell branches and endothelial cell area in comparison to control siRNA treated cells (Fig. 5a–c). VEGF stimulation increased further endothelial cell tube formation in siBMP6-treated cells.

To our surprise, in a matrigel plugin assay in vivo BMP6 was able to induce neovessel formation and infiltration of cells in comparison to control group (Fig. 5d–h). Multiple different cell types were shown to be present in the BMP6 plugs observed by HE-staining, including endothelial cells, immune cells and adipocytes (Fig. 5e). CD31 staining was further used to confirm the presence of endothelial cells and to quantitate neovessel formation (Fig. 5f). Significant increase in the endothelial cell area was seen in the BMP6 group in comparison to control (Fig, 5h). This is to our knowledge the first report showing direct role of BMP6 in angiogenesis in vivo. In comparison to experiments in primary endothelial cells, in tissue BMP6 was observed to induce infiltration of various cell types likely contributing to angiogenesis.

**Fig. 6:**
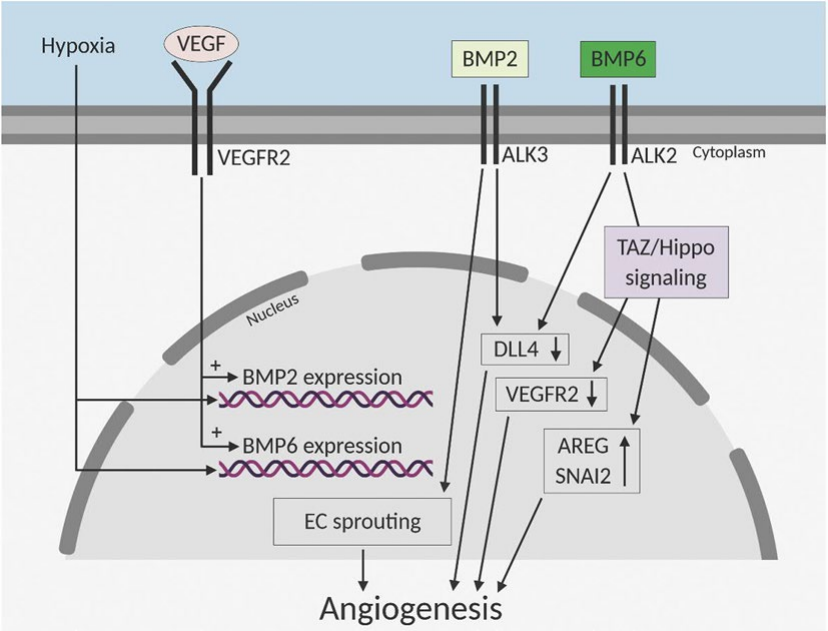
Schematic illustration of BMP/Hippo signaling modulating VEGFR pathway and angiogenic phenotype of endothelial cells. VEGF, bone morphogenetic proteins (BMP) and TAZ-Hippo signaling have synergistic effects on endothelial cell function and sprout formation. Systemic VEGF stimulus, myocardial ischemia, and VEGF stimulus or hypoxia of primary endothelial cells regulate expression of BMPs. Mechanistically, BMP2 and BMP6 modulate VEGF-induced endothelial cell sprouting by regulating expression of tip cell associated genes VEGFR2 or DLL4. BMP6 is able to relocalize TAZ to nucleus and induce subsequent expression of Hippo target genes, and neovessel formation. Illustration was created with BioRender

Altogether, our data demonstrate that various BMP family members are regulated in hypoxia and by VEGF. BMP2 and BMP6 were demonstrated to have an important role in regulating VEGF function. BMP2 induces synergistic effect with VEGF, and acts as a mediator of endothelial sprout formation via regulating expression of DLL4 and VEGFR2. BMP6 instead is able to regulate VEGF-mediated angiogenesis by modulating expression of VEGFR2 via TAZ-Hippo signaling pathway that is an important regulator of cell proliferation. BMP6 protein was further demonstrated, for the first time, to induce angiogenesis in vivo (Fig. 6). Due to their role in regulating VEGFR2 signaling and ability to induce angiogenesis, BMP2/6 are potential targets for anti-angiogenic/pro-angiogenic therapy.

## Discussion

BMP family members are important regulators of both vascular homeostasis and angiogenesis. Synergistic effect of VEGF and BMPs on vasculature have been previously detected in bone formation [53] but their role in angiogenesis, particularly crosstalk with VEGFR2 signaling has remained elusive. Our data demonstrate that BMPs are widely expressed in endothelium of various tissues in hypoxia or normoxia and after VEGF-induced angiogenesis, and that BMP2 and BMP6 regulate VEGFR and Notch signaling. BMP6 was further demonstrated to induce neovessel formation in vivo. This is the first comprehensive data on BMPs in hypoxia, and in angiogenesis in various animal models.

Previously, BMPs have been connected to vascular development including endothelial cell differentiation and venous specification, and to various vascular disorders. For example, in hypoxia-induced pulmonary hypertension, BMP2 expression is increased, leading to upregulation of eNOS, as well as induction of endothelial cell survival and motility via Wnt pathways [54]. Increased BMP6 or BMP2 expression have also been demonstrated in cerebral cavernous malformations and cancer [11, 55, 56]. We show here that VEGF directly regulates transcription of BMP family members -2 and -4 in endothelial cells. Various BMP members, including BMP4 and BMP6 were also regulated in hypoxic endothelial cells. Both BMP2 and BMP6 modulated transcription of VEGFR2 and DLL4 mRNAs, thus regulating VEGF binding to its receptor and tip cell formation. BMP2 was shown to modify the endothelial sprout front, and to increase VEGF-mediated endothelial sprouting. Crosstalk of BMP2 and VEGF in regulating angiogenesis in vivo has been previously published. Synergistic effect of VEGF and BMP2 in angiogenesis was detected in a rabbit model using porous titanium scaffolds loaded with the growth factors [57]. In a xenograft model of hepatoma carcinoma cells, overexpression of BMP2 by virus vectors also increased VEGF transcription and angiogenesis [58]. As we demonstrate that BMP2 is upregulated after VEGF delivery into liver or primary endothelial cells, and that BMP2 modulates VEGFR2 expression and VEGF-mediated endothelial sprouting, our findings support the role of BMP2 in adjusting VEGF-mediated signaling. Since BMP2 was previously suggested to regulate lateral branching of neovessels [59], BMP2 signaling may act as fine-tuning mechanism in VEGF-mediated angiogenesis.

Besides BMP2, other BMPs have been linked to VEGF-mediated angiogenesis prior to this study. Decreased VEGFR2 expression and VE-cadherin internalization was reported with BMP13, leading to stabilization of adherens junctions and increased vascular integrity [21]. BMP4 instead was shown to induce expression and phosphorylation of VEGFR2 [60], and BMP9 to reduce VEGF-mediated angiogenic events in bone-explant angiogenesis assay via an unknown mechanism [61]. In contrast to BMP2, in our study BMP6 showed anti-angiogenic properties in primary endothelial cells, and time-point dependent fluctuation of VEGFR2 expression. To our surprise, instead of anti-angiogenic effects, BMP6 induced neovessel formation and cell infiltration in vivo. The pro-angiogenic properties of BMP6 protein in vivo has not been previously published. We hypothesize that the angiogenic effect caused by BMP6 occurs due to crosstalk with multiple cell types, as these were observed in the plugs, and is thus context dependent. Modulation of angiogenesis by fibroblasts and innate immune cells including macrophages, dendritic cells and mast cells has been previously demonstrated [62, 63]. As BMP6 receptor ALK2 is expressed in multiple cells types besides endothelial cells e.g. in heart and skeletal muscle, BMP6 signaling and crosstalk with other cell types inducing angiogenesis warrants for further studies.

Recently, BMPR pathway was linked for the first time to dysfunctional Hippo-signaling, though the exact extracellular ligands, interaction mechanisms and end-responses remained unknown [46, 48, 64]. We show here that BMP6 induces downstream signaling mediated by Hippo pathway. While active, Hippo signaling retains its downstream effectors YAP/TAZ in the cytoplasm, thus preventing expression of its target genes. While the pathway is inactive, YAP/TAZ is able to translocate to nucleus and induce expression of multiple downstream effectors via TEAD transcription factors, SMADs, p63, RUNX, and PAX [45]. Hippo pathway has previously been shown to regulate multiple cellular functions such as cell proliferation, survival, differentiation, migration and apoptosis. Dysregulation of the pathway has also been linked to cancer metastasis, and to epithelioid hemangioendothelioma [65, 66]. In our study, we show that BMP6 translocates TAZ to nucleus, and induces the expression of Hippo target genes, such as a transcriptional repressor SNAI2, a known regulator of angiogenesis [9, 67]. Likewise, TAZ, which activation and expression levels were here regulated by BMP6, has been previously discovered to regulate angiogenic responses in endothelial cells [48]. Based on our data we hypothesize that BMP6 regulates angiogenesis via Hippo/TAZ downstream factors such as pro-angiogenic growth factor AREG and SNAI2. This is the first time that a BMP family member has been shown to act as a direct mediator of TAZ/Hippo signaling pathway regulating shuttling of TAZ to nucleus. In accordance, ID1, a Hippo target protein, was earlier suggested to be upregulated by BMP6 in microvascular cells [68]. TAZ/YAP was also demonstrated to regulate BMP4 expression in zebrafish [69], and indirectly crosstalk with BMP2 signaling pathway [70]. Additionally, BMP2 has been suggested to induce cytoplasmic retention of YAP [71]. Thus, increasing data suggest crosstalk between BMPR and Hippo signaling pathways.

So far, the interplay between YAP/TAZ and the major signaling pathways regulating angiogenesis has remained poorly understood. Recently, YAP/TAZ was shown to induce VEGFR2 recycling to cell surface and to regulate VEGF-mediated developmental angiogenesis [46, 48, 72]. In vitro, overexpression of YAP/TAZ was also shown to repress BMP and Notch target genes, such as BMP targets SMAD6, UNC5B, ID1; and BMP/Notch targets HES1, DLL4 and HEY1 [48]. Our data demonstrate that regulation of VEGFR2 signaling occurs in part via BMP6/TAZ-Hippo signaling pathway. As YAP and TAZ have shown a differential effect on e.g. adherens junction modulation in endothelial cells, their signaling may differ in various tissue contexts and warrants for further studies.

To conclude, BMP6/Hippo signaling and BMP2 acts as regulators of VEGFR2 signaling pathway. Inhibition of BMP protein expression together with VEGF could be beneficial in the treatment of ocular diseases e.g. age-related macular degeneration, known to express excess amounts of VEGF. As BMP6 upregulation has also been shown to mediate onset and progression of cerebral cavernous malformations by inducing BMP and TGFβ-signaling [11], Hippo pathway inhibitors may act as potential molecular targets for this disease.

## Electronic supplementary material

Below is the link to the electronic supplementary material.

(PDF 44 kb)

(PDF 953 kb) **Supplementary Fig. 1** VEGF expression induce sinusoidal remodeling and an increase of the capillary area. **a** Majority of VEGF mRNA was detected in liver, and minority in lung and heart by RT-qPCR (*n* = 5 animals/group, d6, i.v.). *Ρ*-values are presented between liver and other tissues. **b** VEGF protein levels in plasma and liver d6 after the gene transfer (*n* = 5 animals/group). **c** Quantitation of CD31 positive vessels by immunohistochemistry showed a significant increase in the vascular area of VEGF-treated animals at 6 days after gene transfer (4 images/animal, *n* = 5–10 animals/group). Quantitation was performed by NIS-Elements. **d** Representative images of increased VEGF expression in liver sinusoidal areas (arrowhead) after VEGF gene transfer (i.v., d6) in comparison to AdCMV control group, in which endogenous VEGF expression is limited to areas surrounding portal and central veins (arrow; scale bar 100 µm). **e** Representative images of CD31 stained liver sections showing increased vascular area at d6 after VEGF gene transfer (i.v.; scale bar 100 µm). *Ρ*-values < 0.05*, < 0.01**, < 0.001***. **Supplementary Fig. 2** BMPs and BMP receptors are expressed in several mouse cell types in liver and heart. **a**–**f** Mouse single-cell RNA-sequencing (scRNA-Seq) data from Tabula Muris was used to study the expression of BMP ligands and their receptors in liver and heart. **a** Expression of BMPs 1–9 in liver endothelial cells (EC) is presented, 98% of liver ECs expressed BMP2 and 33% BMP6. **b** Expression of BMPs 1–9 in heart ECs, 27% of ECs expressed BMP6. **c** and **d** Expression of BMP2/4/6 in ECs, fibroblasts (FB), smooth muscle cells (SMC), hepatocytes (HC) and cardiac muscle cells (CM). In liver, BMPs were only expressed in ECs and HCs. In heart, expression of BMPs was detected in various cell types. **e** and **f** Expression levels of specific BMP receptors able to bind BMP2 and BMP6 in liver (**e**) and heart (**f**). All receptors were expressed in several cell types in both tissues, including ECs. In all images, the percentage of gene expressing cells from a cell type specific population is presented. **Supplementary Fig. 3** Role of BMP2/4/6 in regulating EC sprouting in a fibrin bead assay. **a–c** Fibrin bead assay at d7 consisting of both endothelial cells and fibroblasts. HUVECs were grown on collagen-coated beads and embedded on a fibrin gel. HPF cells were put on top of the fibrin gel to enable lumen formation. BMP stimulations (100 ng/ml) were performed every other day. F-actin was stained to visualize endothelial sprouts (red) and DAPI for nuclei (blue) by confocal microscopy (magnification × 10). **a** Representative images of sprouting angiogenesis in untreated (UT) or BMP protein stimulated cells. **b** Quantitation of endothelial cell sprout amount per bead after BMP stimulation. BMP2 was shown to be pro-angiogenic, whereas no statistically significant effect was observed with BMP4 or BMP6 protein. ImageJ analysis, three separate experiments were performed in triplicates, *n* = 30–33 beads/group. **c** No changes in the mRNA expression levels of VEGF, VEGFR1, NRP1/2 were seen in BMP2 or BMP6 stimulated HUVECs (100 ng/ml, 7 h). RT-qPCR detection. *Ρ*-values < 0.05*, < 0.01**, < 0.001***. **Supplementary Fig. 4** TAZ and TEAD2 regulate endothelial cell tube formation and proliferation. **a** Representative images of in vitro tube formation assay in HUVECs 48 h after siRNA treatment with siTAZ. A decrease in tube formation was detected in comparison to control siRNA (*n* = 6/group). Imaged with Olympus IX71 microscope (Tokyo, Japan, magnification × 4). **b** HUVEC proliferation was significantly decreased by siRNAs against TAZ and TEAD2 detected by CyQUANT® proliferation assay (*n* = 6/group, 72 h). **c** siRNA for TAZ efficiently reduced the protein levels of TAZ in HUVECs. Western blot analysis. **d** Taz protein was seen to upregulate and localize into liver sinusoids both in nucleus (arrowhead) and cytoplasm (arrow) after 3 days of AdVEGF gene transfer (i.v.) in comparison to AdCMV control group. Scale bar 100 µm. **e** Taz and Tead mRNA expression levels were upregulated in AdVEGF treated mice liver detected by RT-qPCR (*n* = 4–5/group, i.v., d6). **f** A representative image of immunoblot of TAZ protein localization in nuclear and cytoplasmic fractions in untreated (UT), VEGF or BMP2 protein stimulated (100 ng/ml, 2 h) HUVECs. Lamin A/C (nucleus) and β-actin (cytoplasm) were used as endogenous protein controls. **g** TAZ protein quantities in cytoplasm (cyt) and nucleus (nuc) after 2 h BMP2 protein stimulation. Western blot analysis, normalization to Lamin A/C (nucleus) or β-actin (cytoplasm) and untreated samples. Three independent experiments were performed in triplicates from each cell treatment. **h** BMP2 or BMP6 protein stimulations (100 ng/ml, 7 h) did not affect Hippo signaling target Cysteine Rich Angiogenic Inducer 61 (CYR61) mRNA expression level in HUVECs. **i** CYR61 mRNA expression level was not altered in siTAZ treated and BMP6 stimulated (100 ng/ml, 7 HUVECs in comparison to cells without protein stimulation. *Ρ*-values < 0.05*, < 0.01**, < 0.001***
