# Supplementary material for: BMP6/TAZ-Hippo signaling modulates angiogenesis and endothelial cell response to VEGF
Source: Angiogenesis. 2020 Oct 6;24(1):129–44. doi: 10.1007/s10456-020-09748-4 (PMC7921060; doi:10.1007/s10456-020-09748-4)
Supplement: Supplementary file 1 — (PDF 44 kb) [file 10456_2020_9748_MOESM1_ESM.pdf]

Article title: BMP6/TAZ-Hippo signaling modulates angiogenesis and endothelial cell response to VEGF

Journal: Angiogenesis

Authors: Pulkkinen HH[1]*, Kiema M. [1]*, Lappalainen JP[1,2], Toropainen A[1], Beter M.[1], Tirronen A[1], Holappa L[1], Niskanen H[1], Kaikkonen MU[1], Ylä-Herttuala S[1,3,4]* and Laakkonen JP[1]*.

Affiliations: [1]A.I. Virtanen Institute for Molecular Sciences, University of Eastern Finland, Kuopio, Finland, [2] Department of Clinical Chemistry, University of Eastern Finland and Eastern Finland Laboratory Centre, [3] Science Service Center, Kuopio University Hospital, Kuopio, Finland, and [4] Gene Therapy Unit, Kuopio University Hospital, Kuopio, Finland, *equal contribution

Address correspondence: Johanna Laakkonen PhD., A.I. Virtanen Institute for Molecular Sciences, University of Eastern Finland, Kuopio, Finland, johanna.p.laakkonen@uef.fi, +358503058453, ORCID: 0000-0002-8556-9727

SUPPLEMENTAL FILES

This document includes following material:

1. **Supplemental Methods**
2. **Supplemental Tables: NGS Experiments A-E**
3. **Supplemental Tables: Major Resources Tables**
4. **Supplemental References**

1. SUPPLEMENTAL METHODS

RNA-Seq Libraries & NGS experiments. Tissue sample collection, tissue homogenization and RNA extraction were done in a similar way as for RT-qPCR experiments. After enrichment by Poly(A)-RNA with MicroPoly(A) Purist Kit, RNA was fragmented using RNA Fragmentation Reagents (Life Technologies) and purified in P-30 column (Bio-Rad, Hercules, CA). Fragmented RNA was further dephosphorylated with PNK (New England Biolabs), heat-inactivated and purified by RNA Clean & Concentrator™-5 kit (Zymo Research Corporation). Poly(A)-tailing and cDNA synthesis was performed as described previously [1]. For reverse transcription an oligo allowing custom barcoding during final amplification was used: /5Phos/GATCGTCGGACTGTAGAACTCTGAAC/iSp18/TCAGACGTGTGCTCTTCCGATCTTTTTTTTTTTTTTTTTTTTVN (IDT, Coralville, IA). Exonuclease I (New England Biolabs) was used to catalyze the removal of excess oligo. The DNA-RNA hybrid was further purified using ChIP DNA clean & Concentrator Kit (Zymo Research Corporation), RNaseH treated and circularized using CircLigase (Epicentre). The libraries were amplified for 10 cycles using the following primers: 5’-AATGATACGGCGACCACCGACAGGTTCAGAGTTCTACAGTCCGACG-3’ and 5’-CAAGCAGAAGACGGCATACGAGATXXXXXXGTGACTGGAGTTCAGACGTGTGCTCTTCCGATCT (barcode XXXXXX-underlined). The final product was ran on Novex 10%-TBE gel, purified and cleaned-up as above. The libraries were sequenced on HiSeq 2000 (Illumina, San Diego, CA) for 50 cycles according to the manufacturer's instructions (GeneCore, EMBL). RNA-Seq data pre-processing and analysis was performed as described in [2]. Each sequencing experiment was normalized to a total of 10^7 uniquely mapped tags and visualized by preparing custom tracks for the UCSC Genome browser. Differentially expressed genes were identified using edgeR [3].

Raw data for H3K27ac ChIP-Seq (ENCSR000CDH) was processed as described in [2] and peak detection was performed with HOMER 4.7 findPeaks.pl program using -histone and -nfr options. Those H3K27ac peaks that were detected in two replicate datasets were selected for analysis. To further narrow the regions to detect motifs for putative regulatory factors, we used DNAse I hypersensitive sites detected from 8-week old mouse liver (downloaded from UCSC Table Browser) and selected those found in two replicate datasets and overlapping with H3K27ac peaks. Finally, those DNAse I hypersensitive sites found within promoters (within 3kb from TSS) of differentially regulated genes or at distal regulatory sites (at least 3kb from nearest TSS, but within 100 kb from differentially regulated gene) were used to search for motifs for regulatory factors. Motif enrichment analysis was performed with HOMER findMotifsGenome.pl program.

Mice RNA-Seq data is available in NCBI Gene Expression Omnibus under accession number GSE82106. A summary of the NGS samples used in the analysis and lists of genes in heatmaps are found in Supplemental files: NGS experiments A-E.

SUPPLEMENTAL FILES: NGS EXPERIMENTS

A. SUMMARY OF THE SAMPLES USED IN SEQUENCING EXPERIMENTS AND ANALYSIS

SAMPLES
mouse_liver-RNA-Seq-AdCMV1
mouse_liver-RNA-Seq-AdCMV2
mouse_liver-RNA-Seq-AdCMV3
mouse_liver-RNA-Seq-AdCMV4
mouse_liver-RNA-Seq-AdCMV5
mouse_liver-RNA-Seq-AdVEGF1
mouse_liver-RNA-Seq-AdVEGF2
mouse_liver-RNA-Seq-AdVEGF3
mouse_liver-RNA-Seq-AdVEGF4
mouse_liver-RNA-Seq-AdVEGF5
Pig_GroSeq_healthy_1
Pig_GroSeq_healthy_2
Pig_GroSeq_healthy_3
Pig_GroSeq_border_1
Pig_GroSeq_border_2
Pig_GroSeq_border_3 genes
Pig_GroSeq_ischemic_1
Pig_GroSeq_ischemic_2
Pig_GroSeq_ischemic_3 genes
HUVEC GRO-seq normoxia replicate 1
HUVEC GRO-seq normoxia replicate 2
HUVEC GRO-seq normoxia replicate 3
HUVEC GRO-seq normoxia replicate 4
HUVEC GRO-seq hypoxia replicate 1
HUVEC GRO-seq hypoxia replicate 2
HUVEC GRO-seq hypoxia replicate 3
HUVEC GRO-seq hypoxia replicate 4

B. GO ANALYSIS: SELECTED VEGF-INDUCED CELLULAR PROCESSES AND SIGNALING PATHWAYS
LIVER, i.v. AdVEGF gene transfer, 6d, C57/Bl6 mice

CELLULAR PROCESSES

TermID	Term	Enrichment	logP	Genes in Term	Target Genes in Term	Total Target Genes	Gene Symbols
GO:0001525	angiogenesis	5.30E-28	-62.81	277	76	1036	TEK,DLL4,ID1,HSPG2,COL4A2,MMRN2,KDR,MFGE8,CD34 HAND2,RASIP1,VASH1,NRXN1,TAL1,PDGFA,ENG,NFATC4, JAG1,CTGF,ITGB3,COL4A1,DAB2IP,TBX20,RAPGEF3,TIE1, ADRA2B,PDGFRB,PLCD1,RAMP2,JAM3,SEMA5A,EDNRA, NRARP,PGF,APOLD1,CCBE1,FLT1,PLXND1,ANXA2,NRP1,ESM1, LAMA5,GJA5,HEY1,EFNB2,STAB2,ECSCR,ACKR3,COL8A1,EGFL7, TGFB2,ETS1,SRPX2,BMPER,E2F7,PTPRB,MCAM,FLT4,MMP2, GDF2,SOX17,ANGPT2,NOS3,CSPG4,VEGFC,VAV3,ELK3,NOTCH4 CALCRL,HOXB3,ROBO4,CXCR4,GPR124,ACVRL1,PIK3R6,SOX18
GO:0016477	cell migration	3.38E-16	-35.62	659	101	1036	TEK,AMOTL1,ID1,HBEGF,KDR,CD248,GIPC1,HAND2,DDR1,PDGFB, PLAT,SEMA6A,ENG,ESAM,TGFBR3,CTGF,ITGB3,COL5A1,DAB2IP, TBX20,TIAM1,PDGFRB,FMNL3,MARK1,COL1A1,LRRK2,SEMA3F, VANGL2,JAM3,GPC6,SEMA5A,MDK,FYN,BAMBI,PTK7,FLT1,LAMB1, KITLG,CXCL14,PLXND1,ARC,NRP1,LAMA5,MEF2C,EFNB2,CTHRC1, JUP,SEMA4C,CD63,ITGA6,EPS8,OPHN1,PECAM1,TGFB2,EFNB1, SLC7A6,PLEKHG5,IL16,SPP1,CCL24,NAV1,GLI3,TNFAIP1,APBB2, SRGAP1,PRKG1,EDNRB,NTN1,ITGA9,SOX17,PF4,CCDC88A,ANGPT2, CSPG4,NOS3,VEGFC,TGFB1,TRPM4,ABI2,PPP1R9B,HES1,VAV3,PODXL2, LAMC1,SNAI1,ADAMTS12,RELN,ELANE,PEAK1,CDK5R1,NR2F1,CXCR4, GPR124,FSCN1,PKN3,ACVRL1,HOXA5,NDN,SOX18,COL1A2,CD34
GO:0010594	regulation of endothelial cell migration	1.02E-13	-29.91	88	29	1036	TEK,VEGFC,TGFB1,ANXA3,DLL4,AMOTL1,MMRN2,PTPRM,KDR,BMP10, VASH1,PDGFB,ETS1,SRPX2,SEMA5A,HDAC9,ZNF580,BMPER,FLT4, ACVRL1,GPR124,CCBE1,KLF4,HDAC7,ITGB3,DAB2IP,GDF2,NRP1,ANGPT2
GO:0001936	regulation of endothelial cell proliferation	1.01E-07	-16.11	86	21	1036	TEK,PTPRM,KDR,VASH1,PDGFB,BMP6,SEMA5A,ZNF580,NRARP,BMP2, ENG,PGF,CCL24,FLT4,ACVRL1,ITGB3,GDF2,EGFL7,NRP1,VASH2,CAV2
GO:0001837	epithelial to mesenchymal transition	8.19E-05	-9.41	51	12	1036	TGFB1,BMP2,HEY2,MSX1,TGFBR3,HEYL,SNAI1,FLNA,TGFB2,LOXL3, FAM101B,HEY1
GO:0001955	blood vessel maturation	1.02E-03	-6.89	8	4	1036	ACVRL1,CDH5,LYL1,MMP2
GO:0006955	immune response	8.15E-21	-46.26	1146	75	315	CLEC4E,PTAFR,AK7,CCL2,SLAMF7,CD274,CCL5,CD1D,GBP5,PSMB8,SOCS1, HLA-DQB1,GBP2,IL1RN,IFIT1,ZBP1,C2,TLR5,IFNG,RIPK2,USP18,SLC26A6, SOCS3,CXCL10,TAPBP,UBD,CFD,STAT1,IRF7,HLA-DRB5,GBP6,CD276,HLA-F, CXCL3,CD3G,CXCL2,IRGM,CD27,ICAM1,HLA-A,CD180,PDCD1,UBE2L6,SLA2, CCL4,AQP4,PYCARD,PRF1,ITK,IL7,RORC,TNF,TNFAIP3,HLA-DQA1,VNN1, CST7,IFIT3,CD74,ISG15,CIITA,FAS,PSMB10,TLR2,CD8A,RSAD2,SEMA4D, CAMK2B,NFIL3,NLRC5,LY75,CCL13,CCL3L3,IRG1,CD28,IRF1
GO:0033993	response to lipid	6.44E-07	-14.26	646	33	315	PTAFR,NQO1,CCL2,WNT2,CCL5,GSTP1,ICAM1,SLC34A2,CD180,CYP1A1, SERPINA7,MAPK15,PYCARD,AQP4,IL1RN,CEBPE,TLR5,IFNG,RORC,TNF, CYP7A1,TNFAIP3,RIPK2,FAS,CXCL10,TLR2,CYP1A2,PTGDS,STAT1,AR,CMPK2, PTGES,IRG1

SIGNALING PATHWAYS

TermID	Term	Enrichment	logP	Genes in Term	Target Genes in Term	Total Target Genes	Gene Symbols
GO:0007179	transforming growth factor beta receptor signaling pathway	9.07E-06	-11.61	129	23	1036	TGFB1,CDKN2B,ID1,SMAD9,TGIF2,SMAD7,EID2,TGFB2,PDGFB,COL3A1, PDGFA,ENG,FKBP1A,TGFBR3,LTBP4,BAMBI,PMEPA1,ACVRL1,WWTR1, TGFB3,COL1A2,SMAD6,PDGFRB
GO:0030509	BMP signaling pathway	6.10E-05	-9.70	65	14	1036	ENG,BMP2,MSX1,ID1,TGFBR3,FSTL1,SMAD9,GDF6,ACVRL1,BMP10, SMAD7,GDF2,SMAD6,BMP6
GO:0007219	Notch signaling pathway	1.83E-04	-8.61	126	20	1036	HES1,DLL4,SNAI1,NOTCH4,DTX3,JAG2,NLE1,APP,HDAC9,CCND1,BMP2, MDK,HEY2,EPN2,JAG1,HEYL,HDAC7,NOTCH3,MAML3,HEY1
GO:0048010	vascular endothelial growth factor receptor signaling pathway	6.35E-04	-7.36	24	7	1036	VEGFC,PGF,DAB2IP,NRP1,FLT4,KDR,FLT1
GO:0071526	semaphorin-plexin signaling pathway	1.76E-03	-6.34	21	6	1036	PLXNA4,SEMA3F,PLXND1,NRP1,SEMA4C,SEMA6A
GO:0035329	hippo signaling	5.64E-03	-5.18	26	6	1036	TJP1,AMOTL1,FAT4,TEAD2,WWTR1,DCHS1
GO:0016055	Wnt signaling pathway	2.03E-02	-3.90	236	24	1036	TNIK,CTHRC1,AMOTL1,MARK1,JUP,WLS,WISP1,NXN,DACT3,HIC1,SOX4, VANGL2,DKK2,CCND1,TMEM88,DAB2,FRZB,PTK7,CDH3,TAX1BP3,CCDC88C, SOX17,RARG,TGFB1I1

C. DIFFERENTIALLY REGULATED GENES: TGF-beta/BMP SIGNALING
LIVER, i.v. AdVEGF gene transfer, 6d, C57/Bl6 mice

Transcript/RepeatID	Chr	Strand	Symbol	Description	AdCMV vs. AdVEGF logFC	AdCMV vs. AdVEGF FDR
NM_001277259	chr15	+	Alk1/Acvrl1	activin A receptor, type II-like 1	1.28	1.7E-08
NM_175501	chr15	+	Adamts12	a disintegrin-like and metallopeptidase (reprolysin type) with thrombospondin type 1 motif, 12	2.50	8.3E-13
NM_026505	chr18	+	Bambi	BMP and activin membrane-bound inhibitor	1.45	3.9E-07
NM_007553	chr2	+	Bmp2	bone morphogenetic protein 2	1.70	2.1E-13
NM_007556	chr13	+	Bmp6	bone morphogenetic protein 6	2.07	3.0E-04
NM_019506	chr14	+	Bmp9/Gdf2	growth differentiation factor 2	1.03	7.7E-05
NM_009756	chr6	+	Bmp10	bone morphogenetic protein 10	1.81	1.7E-02
NM_013526	chr4	+	Bmp13/Gdf6	growth differentiation factor 6	1.14	2.5E-02
NM_028472	chr9	+	Bmper	BMP-binding endothelial regulator	1.31	1.4E-02
NM_016900	chr6	+	Cav2	caveolin 2	1.46	4.3E-10
NM_153098	chr9	+	Cd109	CD109 antigen	6.72	2.9E-12
NM_007670	chr4	-	Cdkn2b	cyclin dependent kinase inhibitor 2B	1.92	2.1E-03
NM_007742	chr11	+	Col1a1	collagen, type I, alpha 1	3.15	2.8E-18
NM_007743	chr6	+	Col1a2	collagen, type I, alpha 2	3.02	5.2E-20
NM_009930	chr1	+	Col3a1	collagen, type III, alpha 1	2.67	9.3E-24
NM_009932	chr8	+	Col4a2	collagen, type IV, alpha 2	2.71	5.9E-30
NM_001037905	chr15	+	Dab2	disabled 2, mitogen-responsive phosphoprotein	1.49	1.9E-16
NM_198425	chr7	+	Eid2	EP300 interacting inhibitor of differentiation 2	2.01	2.8E-05
NM_001146350	chr2	+	Eng	endoglin	1.42	9.8E-14
NM_007993	chr2	-	Fbn1	fibrillin 1	2.49	4.6E-28
NM_008019	chr2	+	Fkbp1a	FK506 binding protein 1a	1.11	1.6E-06
NM_008047	chr16	+	Fstl1	follistatin-like 1	2.67	4.4E-32
NM_018771	chr8	+	Gipc1	GIPC PDZ domain containing family, member 1	1.37	1.1E-10
NM_008235	chr16	+	Hes1	hes family bHLH transcription factor 1	1.79	5.7E-14
NM_013905	chr4	+	Heyl	hairy/enhancer-of-split related with YRPW motif-like	3.48	1.9E-31
NM_019564	chr7	+	Htra1	HtrA serine peptidase 1	1.22	1.5E-02
NM_030127	chr5	-	Htra3	HtrA serine peptidase 3	1.53	1.3E-02
NM_010495	chr2	+	Id1	inhibitor of DNA binding 1	2.46	3.9E-28
NM_001001309	chr2	-	Itga8	integrin alpha 8	1.68	1.7E-08
NM_001113549	chr7	-	Ltbp4	latent transforming growth factor beta binding protein 4	2.36	1.0E-19
NM_001291035	chr11	-	Mapk7	mitogen-activated protein kinase 7	1.29	1.5E-06
NM_007963	chr3	-	Mecom	MDS1 and EVI1 complex locus	4.54	4.8E-81
NM_001170537	chr13	+	Mef2c	myocyte enhancer factor 2C	3.02	3.7E-46
NM_010835	chr5	-	Msx1	msh homeobox 1	1.85	1.5E-03
NM_001109988	chr18	-	Nrep	neuronal regeneration related protein	1.13	6.2E-05
NM_001243757	chr7	+	Pde2a	phosphodiesterase 2A, cGMP-stimulated	1.69	3.0E-21
NM_018779	chr6	+	Pde3a	phosphodiesterase 3A, cGMP inhibited	1.83	9.0E-05
NM_008808	chr5	-	Pdgfa	platelet derived growth factor, alpha	1.62	1.9E-14
NM_011057	chr15	-	Pdgfb	platelet derived growth factor, B polypeptide	2.28	6.6E-12
NM_008809	chr18	+	Pdgfrb	platelet derived growth factor receptor, beta polypeptide	1.82	8.4E-17
NM_001040611	chr6	+	Peg10	paternally expressed 10	2.85	6.3E-13
NM_022995	chr2	-	Pmepa1	prostate transmembrane protein, androgen induced 1	1.62	4.9E-15
NM_008542	chr9	-	Smad6	SMAD family member 6	1.71	5.7E-14
NM_001042660	chr18	+	Smad7	SMAD family member 7	1.26	2.4E-08
NM_019483	chr3	+	Smad9	SMAD family member 9	1.41	2.5E-04
NM_009234	chr12	-	Sox11	SRY (sex determining region Y)-box 11	6.27	3.9E-28
NM_011577	chr7	+	Tgfb1	transforming growth factor, beta 1	1.46	2.1E-03
NM_001289553	chr7	+	Tgfb1i1	transforming growth factor beta 1 induced transcript 1	2.13	1.7E-08
NM_009367	chr1	-	Tgfb2	transforming growth factor, beta 2	2.79	1.1E-09
NM_009368	chr12	-	Tgfb3	transforming growth factor, beta 3	3.45	2.1E-14
NM_011578	chr5	-	Tgfbr3	transforming growth factor, beta receptor III	1.24	8.8E-06
NM_001291124	chr2	+	Tgif2	TGFB-induced factor homeobox 2	2.12	7.7E-04
NM_133784	chr3	-	Wwtr1	WW domain containing transcription regulator 1	1.41	4.4E-13

D. DIFFERENTIALLY REGULATED GENES: TGF-beta/BMP SIGNALING
MYOCARDIUM, domestic pig, ischemia samples
GSE81155

Transcript/RepeatID	Chr	Strand	Symbol	healthy vs ischemic logFC	healthy vs ischemic FDR	healthy vs border logFC	healthy vs border FDR	border vs ischemic logFC	border vs ischemic FDR
NM_001204765	chr15	-	ACVR2A	-1.21	6.02E-04	-0.68	2.87E-02	-0.41	3.66E-01
NM_001204901_1	chr7	-	BMP5	-3.26	3.56E-08	-0.91	1.37E-02	-2.19	7.91E-04
NM_001099926	chr3	+	ADAM17	1.26	1.40E-04	0.91	5.90E-03	0.23	1.00E+00
NM_214048	chr1	-	ARG1	7.62	3.27E-48	6.91	4.29E-54	0.85	3.47E-03
NM_001243889	chr3	-	ASPN	-2.87	1.68E-10	-0.96	4.92E-03	-1.75	1.10E-03
NM_001195399	chr17	+	BMP2	6.38	5.13E-05	4.07	5.75E-02	2.10	9.33E-03
NM_001101031	chr1	-	BMP4	1.01	2.06E-01	-0.69	5.56E-01	1.80	1.56E-02
NM_001105290	chr17	-	BMP7	3.94	3.44E-02	3.19	4.46E-02	0.71	8.17E-01
NM_001204767	chr14	+	BMPR1A	-1.51	1.92E-03	-0.26	7.42E-01	-1.14	3.31E-02
NM_214438	chr18	-	CAV1	-0.85	1.98E-02	-0.65	3.66E-02	-0.08	1.00E+00
NM_001123091	chr18	-	CAV2	-1.06	5.58E-02	-0.76	8.96E-02	-0.15	1.00E+00
NM_214214	chr12	+	CCL2	3.42	4.68E-12	2.58	4.30E-11	0.91	3.27E-02
NM_001037964	chrX	-	CITED1	-2.69	1.60E-02	-1.34	1.18E-01	-1.16	8.17E-01
NM_001037965	chr3	-	ID2	2.12	1.50E-04	2.57	1.75E-14	-0.34	7.93E-01
NM_001128444	chr4	-	F11R	1.25	6.84E-03	0.32	7.83E-01	0.97	1.16E-01
NM_001123113	chr7	+	FOS	2.69	1.89E-16	2.56	5.13E-20	0.21	8.42E-01
NM_213899	chr7	+	FUT8	1.08	1.66E-03	1.17	9.88E-06	0.04	1.00E+00
NM_214293	chr14	+	GATA4	-2.19	5.15E-10	-0.64	7.46E-02	-1.47	9.47E-10
NM_214328	chr6	+	GATA6	-3.04	7.04E-12	-1.26	1.13E-05	-1.68	1.04E-11
NM_001123124	chr1	+	HIF1A	1.08	1.71E-03	0.83	5.30E-03	0.34	5.67E-01
NM_001190248	chr14	+	HPGD	-1.31	5.06E-02	-1.16	1.37E-02	0.02	1.00E+00
NM_001134344	chr4	-	KLF10	1.06	3.24E-02	0.90	1.42E-02	0.27	9.09E-01
NM_001129967	chr8	+	LEF1	1.88	4.48E-04	0.65	4.06E-01	1.34	1.81E-02
NM_001114280	chr1	-	MAP3K7	-0.83	3.78E-02	-0.39	4.24E-01	-0.36	6.92E-01
NM_214435	chr15	-	MSTN	-6.31	2.33E-02	-2.25	1.53E-01	-4.41	6.20E-01
NM_001005154	chr4	-	MYC	2.19	6.58E-08	1.22	1.42E-03	1.06	1.60E-03
NM_213736	chr5	-	PDE3A	-3.60	1.08E-10	-1.50	4.80E-07	-1.99	3.48E-12
NM_001123203	chr17	-	PMEPA1	1.29	4.30E-04	1.06	4.08E-04	0.33	6.02E-01
NM_001044560	chr14	-	PPP1CC	-0.99	3.67E-02	-0.09	1.00E+00	-0.78	1.59E-01
NM_214321	chr9	-	PTGS2	3.39	1.36E-13	1.99	3.71E-05	1.51	6.28E-05
NM_001145750_1	chr14	+	SIRT1	-2.12	6.87E-03	-0.17	1.00E+00	-1.87	2.47E-02
NM_001195510	chr11	-	SMAD9	0.74	4.90E-01	1.38	4.95E-03	-0.56	7.34E-01
NM_214015_1	chr6	-	TGFB1	1.33	6.64E-04	1.09	5.58E-04	0.35	6.28E-01
NM_001244536	chr1	-	THBS1	1.37	4.02E-03	0.93	1.82E-02	0.54	2.45E-01
NM_001097436	chrX	+	XIAP	-1.20	3.77E-03	-0.76	2.73E-02	-0.36	7.89E-01

*not significant in the comparison

E. DIFFERENTIALLY REGULATED GENES IN HYPOXIC ENDOTHELIAL CELLS: TGF-beta/BMP PRODUCTION OR SIGNALING
HUVEC cells: normoxia, hypoxia
GSE94872

Transcript/RepeatID	Chr	Strand	Symbol	notx vs. hypox logFC	notx vs. hypox FDR
NM_130851	chr14	-	BMP4	2.02	1.25E-33
NM_002982	chr17	+	CCL2	0.98	1.56E-04
NM_003278	chr3	+	CLEC3B	1.74	4.48E-08
NM_000088	chr17	-	COL1A1	1.45	2.28E-03
NM_001554	chr1	+	CYR61	0.82	1.16E-11
NM_001951	chr8	+	E2F5	-1.19	7.64E-08
NM_001964	chr5	+	EGR1	-1.82	5.74E-04
NR_038170	chr14	+	FUT8	-1.17	3.10E-24
NM_003505	chr7	+	FZD1	1.34	8.26E-03
NM_005524	chr3	+	HES1	1.04	4.33E-10
NM_001530	chr14	+	HIF1A	-0.84	1.06E-08
NM_022740	chr7	-	HIPK2	-0.81	2.50E-13
NM_001297559	chr4	+	HTRA3	0.86	1.65E-05
NM_002228	chr1	-	JUN	0.90	6.42E-14
NM_001166265	chr2	+	LTBP1	-0.93	3.38E-13
NM_001177413	chr2	+	RPS27A	-0.84	5.56E-08
NM_001190822	chr18	-	SMAD7	0.91	5.32E-11
NM_003244	chr18	+	TGIF1	0.77	4.86E-07
NM_138440	chr16	+	VASN	1.98	1.56E-05
NM_002166	chr2	+	ID2	1.14	1.88E-06
NM_001001557	chr8	-	GDF6/BMP13	2.32	2.52E-16

3. Major Resources Tables

Animals

Species	Vendor or Source	Background Strain	Sex	Age
Mice	Harlan Laboratories	C57/Bl6	male	8-12 weeks
Mice	Envigo	Hsd:Athymic Nude-*Foxn1*^{nu}	female	6 weeks

Animal breeding

	Species	Vendor or Source	Background Strain	Other Information
**Parent - Male**	Mice	Harlan Laboratories	C57/Bl6	breeding in the Lab Animal Unit of the University of Eastern Finland
**Parent - Female**	Mice	Harlan Laboratories	C57/Bl6	breeding in the Lab Animal Unit of the University of Eastern Finland

Antibodies

Target antigen	Vendor or Source	Catalog #	Working concentration
Alexa 488 chicken anti-rabbit	Thermo Fisher Scientific	#A21441	IF: 1/200
Beta-actin	Cell Signaling Technology	#4967	WB: 1/1000
BMP2	LS BioSciences	#LS-C407899	IF: 1/500
BMP6	Thermo Fisher Scientific	#PA5-78879	WB: 1/2000
CD31	BD Biosciences	#550274	IHC: 1/50
Phalloidin Alexa-635	Thermo Fisher Scientific	#A2287	IF: 1/40
pTAZ (Ser89)	Cell Signaling Technology	#59971	WB: 1/1000
Lamin A/C (4C11)	Cell Signaling Technology	#4777	WB: 1/1000
VEGF	Abcam	#52917	IHC: 1/100
VEGFR2 (55B11)	Cell Signaling Technology	#2479	WB: 1/1000, IF: 1/50
YAP/TAZ	Cell Signaling Technology	#8418	WB: 1/1000
TAZ	Sigma Aldrich	# HPA007415	IHC: 1/100

Cultured Cells

Name	Vendor or Source	Sex (F, M, or unknown)
HUVEC	Kuopio University Hospital	F
HPF-C	Promocell	unknown

Primers

Gene	Full name	Assay ID	Vendor
mBmp2	Bone Morphogenetic Protein 2 (mouse)	qMmuCIP0030212	BioRad
mBmp4	Bone Morphogenetic Protein 4 (mouse)	qMmuCEP0054665	BioRad
mBmp6	Bone Morphogenetic Protein 6 (mouse)	qMmuCIP0032439	BioRad
mBmp10	Bone Morphogenetic Protein 10 (mouse)	qMmuCIP0028954	BioRad
mGdf2	Growth Differentiation Factor 2 (BMP9) (mouse)	qMmuCIP0029928	BioRad
mGdf6	Growth Differentiation Factor 6 (BMP13) (mouse)	qMmuCIP0031409	BioRad

mPdgfb	Platelet-Derived Growth Factor Beta Polypeptide (mouse)	Mm00440677_m1	Thermo Fisher Scientific
mPpia	Peptidylprolyl Isomerase A (mouse)	Mm03302254_g1	Thermo Fisher Scientific
mTead2	TEA Domain Transcription Factor 2 (mouse)	Mm00449004_m1	Thermo Fisher Scientific
mTgfb1	Transforming Growth Factor Beta 1 (mouse)	Mm01178820_m1	Thermo Fisher Scientific
mTgfb3	Transforming Growth Factor Beta 3 (mouse)	Mm00436960_m1	Thermo Fisher Scientific
mVegfa	Vascular Endothelial Growth Factor A (mouse)	Mm01281449_m1	Thermo Fisher Scientific
mWwtr1	WW Domain Containing Transcription Regulator 1 (mouse)	Mm01289583_m1	Thermo Fisher Scientific
hAREG	Amphiregulin (human)	qHsaCIP0039790	BioRad
hB2M	Beta-2-Microglobulin (human)	qHsaCIP0029872	BioRad
hBMP2	Bone Morphogenetic Protein 2 (human)	qHsaCIP0029912	BioRad
hBMP4	Bone Morphogenetic Protein 4 (human)	qHsaCEP0024625	BioRad
hBMP6	Bone Morphogenetic Protein 6 (human)	qHsaCIP0031092	BioRad
hCTGF	Connective Tissue Growth Factor (human)	qHsaCEP0024255	BioRad
hCYR61	Cysteine Rich Angiogenic Inducer 61 (human)	qHsaCEP0024230	BioRad
hDLL4	Delta-Like 4 (human)	qHsaCEP0051500	BioRad
hFLT1	Fms Related Tyrosine Kinase 1 (VEGFR1) (human)	Hs01052961_m1	Thermo Fisher Scientific
hKDR	Kinase Insert Domain Receptor (VEGFR2) (human)	Hs00911700_m1	Thermo Fisher Scientific
hNRP1	Neuropilin 1 (human)	Hs00826128_m1	Thermo Fisher Scientific
hNRP2	Neuropilin 2 (human)	Hs00187290_m1	Thermo Fisher Scientific
hSNAI2	Snail Family Transcriptional Repressor 2 (human)	qHsaCIP0027976	BioRad
hTEAD2	TEA Domain Transcription Factor 2 (human)	qHsaCIP0030996	BioRad
hVEGFA	Vascular Endothelial Growth Factor A (human)	Hs00900055_m1	Thermo Fisher Scientific
hWWTR1	WW Domain Containing Transcription Regulator 1 (human)	qHsaCEP0049274	BioRad

Recombinant proteins

**Protein**	**Vendor or Source**	**Catalog #**	**Working concentration**
BMP2	R&D Systems	#355-BM-010	100 ng/ml
BMP4	R&D Systems	#314-BP-010	100 ng/ml
BMP6	R&D Systems	#507-BP-020	100 ng/ml
VEGF	R&D Systems	#293-VE-010	100 ng/ml

siRNAs

**siRNA**	**Vendor or Source**	**Catalog #**	**Working concentration**
Negative Control 2 siRNA	Thermo Fisher Scientific	#4390846	5/10 nM
siTAZ, s24788	Thermo Fisher Scientific	#4392420	5/10 nM
siBMP2, s2020	Thermo Fisher Scientific	#4392420	5 nM
siBMP6, s2034	Thermo Fisher Scientific	#4392420	5 nM

4. SUPPLEMENTAL REFERENCES:

1. Wang D, Garcia-Bassets I, Benner C, et al. Reprogramming transcription by distinct classes of enhancers functionally defined by eRNA. Nature. 2011;474(7351):390-394.

2. Niskanen H, Tuszynska I, Zaborowski R, et al. Endothelial cell differentiation is encompassed by changes in long range interactions between inactive chromatin regions. *Nucleic Acids Res*. 2018;46(4):1724-1740.

3. Robinson MD, McCarthy DJ, Smyth GK. edgeR: A bioconductor package for differential expression analysis of digital gene expression data. Bioinformatics. 2010;26(1):139-140.
