# Supplementary figures and images for: BMP6/TAZ-Hippo signaling modulates angiogenesis and endothelial cell response to VEGF
Source: Angiogenesis. 2020 Oct 6;24(1):129–44. doi: 10.1007/s10456-020-09748-4 (PMC7921060; doi:10.1007/s10456-020-09748-4)

# Suppl. Fig. 1

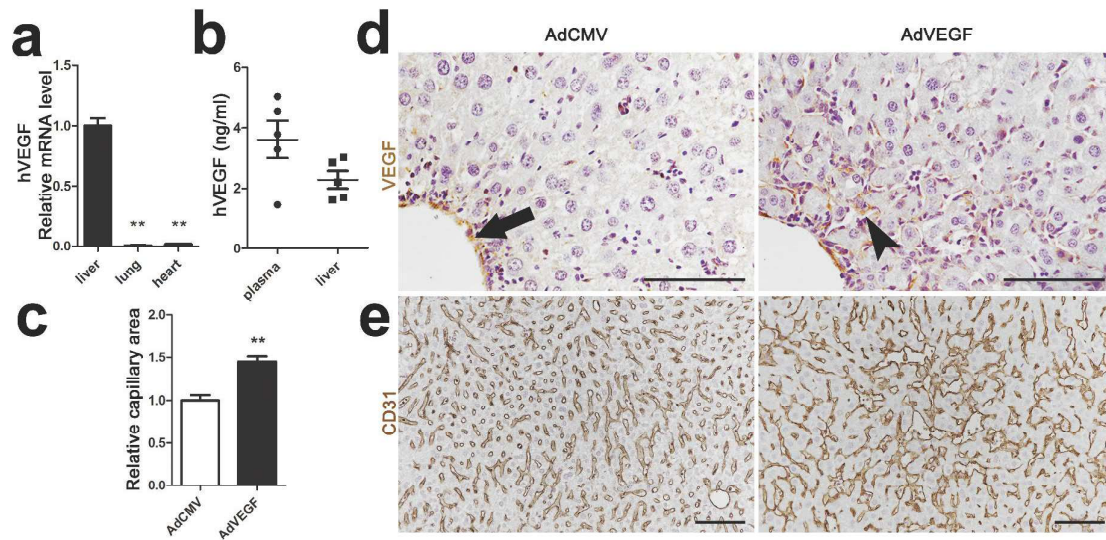

# Suppl. Fig. 2

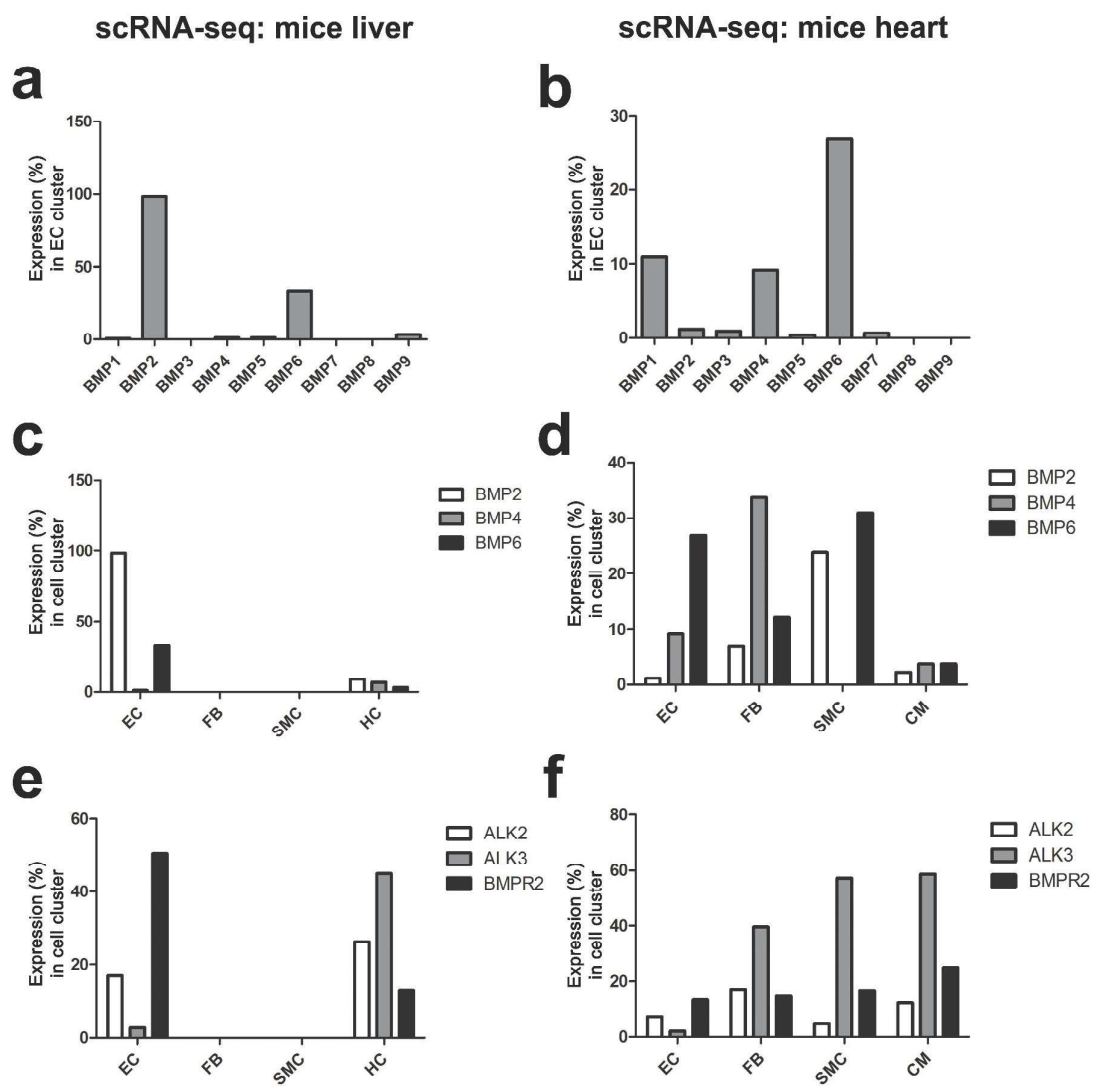

# Suppl. Fig. 3

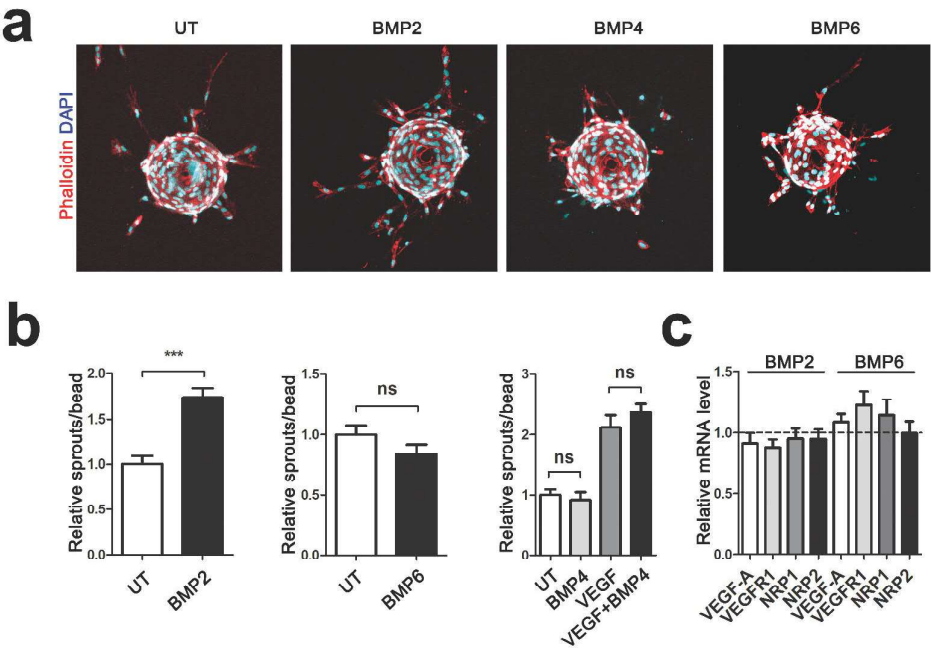

# Suppl. Fig. 4

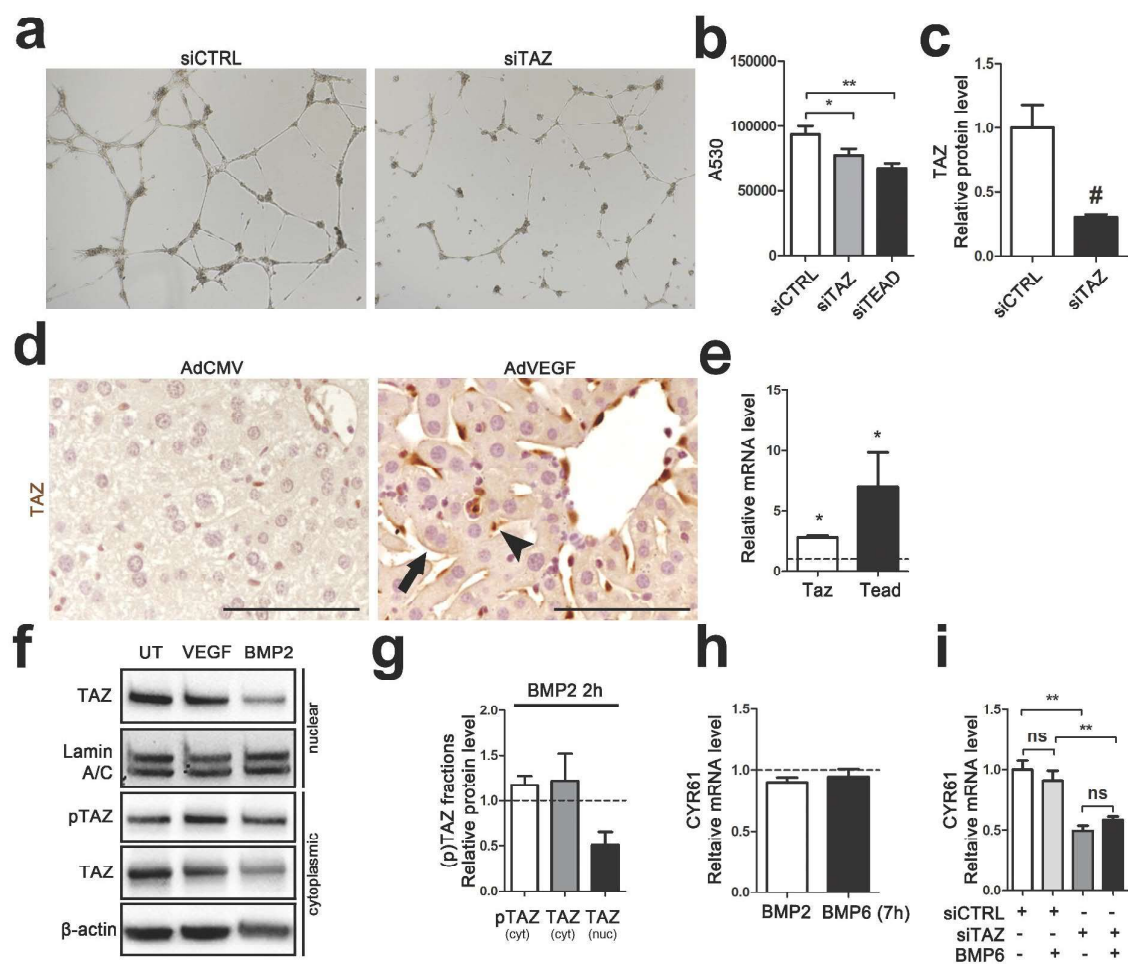

Supplement: Supplementary file 2 — (PDF 953 kb) Supplementary Fig. 1 VEGF expression induce sinusoidal remodeling and an increase of the capillary area. a Majority of VEGF mRNA was detected in liver, and minority in lung and heart by RT-qPCR (n = 5 animals/group, d6, i.v.). Ρ-values are presented between liver and other tissues. b VEGF protein levels in plasma and liver d6 after the gene transfer (n = 5 animals/group). c Quantitation of CD31 positive vessels by immunohistochemistry showed a significant increase in the vascular area of VEGF-treated animals at 6 days after gene transfer (4 images/animal, n = 5–10 animals/group). Quantitation was performed by NIS-Elements. d Representative images of increased VEGF expression in liver sinusoidal areas (arrowhead) after VEGF gene transfer (i.v., d6) in comparison to AdCMV control group, in which endogenous VEGF expression is limited to areas surrounding portal and central veins (arrow; scale bar 100 µm). e Representative images of CD31 stained liver sections showing increased vascular area at d6 after VEGF gene transfer (i.v.; scale bar 100 µm). Ρ-values < 0.05*, < 0.01**, < 0.001***. Supplementary Fig. 2 BMPs and BMP receptors are expressed in several mouse cell types in liver and heart. a–f Mouse single-cell RNA-sequencing (scRNA-Seq) data from Tabula Muris was used to study the expression of BMP ligands and their receptors in liver and heart. a Expression of BMPs 1–9 in liver endothelial cells (EC) is presented, 98% of liver ECs expressed BMP2 and 33% BMP6. b Expression of BMPs 1–9 in heart ECs, 27% of ECs expressed BMP6. c and d Expression of BMP2/4/6 in ECs, fibroblasts (FB), smooth muscle cells (SMC), hepatocytes (HC) and cardiac muscle cells (CM). In liver, BMPs were only expressed in ECs and HCs. In heart, expression of BMPs was detected in various cell types. e and f Expression levels of specific BMP receptors able to bind BMP2 and BMP6 in liver (e) and heart (f). All receptors were expressed in several cell types in both tissues, including ECs. [file 10456_2020_9748_MOESM2_ESM.pdf]
